# A phylogeny and molecular barcodes for *Caenorhabditis*, with numerous new species from rotting fruits

**DOI:** 10.1186/1471-2148-11-339

**Published:** 2011-11-21

**Authors:** Karin C Kiontke, Marie-Anne Félix, Michael Ailion, Matthew V Rockman, Christian Braendle, Jean-Baptiste Pénigault, David HA Fitch

**Affiliations:** 1 Department of Biology, New York University, 100 Washington Square East New York, New York 10003, USA; 2 CNRS-Institut Jacques Monod, 15 rue Hélène Brion, 75205 Paris cedex 13, France; 3 Department of Biology, University of Utah, Salt Lake City, Utah 84112, USA; 4 Center for Genomics and Systems Biology, New York University, New York, USA; 5 Institute of Developmental Biology and Cancer, CNRS-University of Nice, Sophia-Antipolis, Parc Valrose, 06108 NICE cedex 2, France; 6 Department of Biochemistry, University of Washington, Seattle, WA, USA

## Abstract

**Background:**

The nematode *Caenorhabditis elegans *is a major laboratory model in biology. Only ten *Caenorhabditis *species were available in culture at the onset of this study. Many of them, like *C. elegans*, were mostly isolated from artificial compost heaps, and their more natural habitat was unknown.

**Results:**

*Caenorhabditis *nematodes were found to be proliferating in rotten fruits, flowers and stems. By collecting a large worldwide set of such samples, 16 new *Caenorhabditis *species were discovered. We performed mating tests to establish biological species status and found some instances of semi-fertile or sterile hybrid progeny. We established barcodes for all species using ITS2 rDNA sequences. By obtaining sequence data for two rRNA and nine protein-coding genes, we determined the likely phylogenetic relationships among the 26 species in culture. The new species are part of two well-resolved sister clades that we call the *Elegans *super-group and the *Drosophilae *super-group. We further scored phenotypic characters such as reproductive mode, mating behavior and male tail morphology, and discuss their congruence with the phylogeny. A small space between rays 2 and 3 evolved once in the stem species of the *Elegans *super-group; a narrow fan and spiral copulation evolved once in the stem species of *C. angaria*, *C*. sp. 8 and *C*. sp. 12. Several other character changes occurred convergently. For example, hermaphroditism evolved three times independently in *C. elegans*, *C. briggsae *and *C*. sp. 11. Several species can co-occur in the same location or even the same fruit. At the global level, some species have a cosmopolitan distribution: *C. briggsae *is particularly widespread, while *C. elegans *and *C. remanei *are found mostly or exclusively in temperate regions, and *C. brenneri *and *C*. sp. 11 exclusively in tropical zones. Other species have limited distributions, for example *C*. sp. 5 appears to be restricted to China, *C*. sp. 7 to West Africa and *C*. sp. 8 to the Eastern United States.

**Conclusions:**

*Caenorhabditis *are "fruit worms", not soil nematodes. The 16 new species provide a resource and their phylogeny offers a framework for further studies into the evolution of genomic and phenotypic characters.

## Background

The nematode worm *Caenorhabditis elegans *is a key laboratory model system which has provided key insights into molecular biology (e.g. RNA interference and small RNAs), cell biology (cell polarity, apoptosis), developmental biology (signal transduction pathways, developmental timing) and neurobiology (axon guidance, synaptic function). The use of *C. elegans *and related species for evolutionary biology has recently increased [see [1-9]]. Several characteristics make this roundworm an interesting species for evolutionary studies, among them the accumulated knowledge on its biology, its simplicity of use (including the ability to cryogenically preserve living strains) and its selfing mode of reproduction with facultative outcrossing.

However, *C. elegans *lacks an extensive evolutionary framework of closely related species, especially when compared to *Drosophila melanogaster*. Ten *Caenorhabditis *species were available in culture prior to this work, compared with over 2000 described *Drosophila *species [10]. More *Caenorhabditis *species are known from the literature [11,12] but they have not been re-isolated and are thus not available for further studies. In addition, molecular divergence among *Caenorhabditis *species is greater than among *Drosophila *species [3]. For example *C. elegans *and *C. briggsae*, regarded as close relatives within *Caenorhabditis*, are probably as distant as *D. melanogaster *and *D. ananassae*, regarded as fairly distant relatives within *Drosophila *[13,14]. The tiny size of these animals and the small number of taxonomists focusing on this taxonomic group may explain in great part the paucity of described species.

A key additional reason for the lack of known diversity in the *Caenorhabditi*s genus is that these so-called "soil nematodes" are rarely found in soil samples. Soil samples yield a variety of nematode species, including a few species of the family Rhabditidae (to which *Caenorhabditis *belongs). For example, some *Oscheius *species are readily found in soil samples [15]. However, despite extensive sampling for many years, we failed to isolate *Caenorhabditis *from soil [16]. Rare positive instances correspond to soil of orchards (e.g. *C. elegans *strain JU258, Madeira 2001), or soil below trees with rotting fruits (*C*. sp. 5 JU727). Instead, *C. elegans*, *C. briggsae *and *C. remanei *were found in compost heaps containing decaying vegetal material [11,17].

We screened rotting vegetal material for the presence of *Caenorhabditis *and found that *Caenorhabditis *species are most readily isolated from rotting fruits and flowers, and occasionally from other rotten plant parts (e.g. banana pseudo-stems, but rarely leaves). Focusing on rotten fruit samples, we found a number of new isolates of *C. elegans*, *C. briggsae*, *C. remanei *and *C. brenneri*. In addition, we collected sixteen new *Caenorhabditis *species, which dramatically increased the number of *Caenorhabditis *species presently in culture to 26. We obtained sequences of SSU and LSU rRNA genes and of nine protein-coding genes in these new isolates and built an expanded molecular phylogeny of the genus *Caenorhabditis*. We tested the new species for reproductive isolation by pairwise mating tests. Finally, we explored the ITS regions of the rRNA gene array for their suitability as a barcode and found that a specimen can reliably be assigned to one of the *Caenorhabditis *species in culture using the ITS2 sequence. Finally, we report on the evolution of a number of phenotypic characters in the genus, including the mode of reproduction.

## Methods

### Sampling and isolation

Rotting vegetal material was sampled and stored in tubes or plastic bags. Care was taken during storage and transport to provide some oxygen to the samples, and excessive heat was avoided. *Caenorhabditis *individuals could be retrieved after up to three weeks of sample storage.

Collected samples were placed in the laboratory on standard *C. elegans *culture plates seeded in the center with the *E. coli *OP50 strain. The samples were deposited outside the bacterial lawn and humidified by addition of one to three milliliters of water or M9 buffer [18]. *Caenorhabditis *nematodes are attracted by the *E. coli *lawn and tend to remain on the lawn, often along the thicker lawn edge. They can often be recognized with a dissecting microscope equipped with transillumination (40-50×) by a combination of morphological criteria: the color of their intestine (light brown), the large intestinal cell nuclei visible as white disks on the brown intestinal cytoplasm background, the long and fine tail of the adult hermaphrodite, the vulva position in the center of the animal, and the short and round tail tip in the adult male [18]. Using a compound microscope equipped with differential interference contrast, further scorable characters are the presence of a round median pharyngeal bulb, the characteristic vulva cell division pattern [2], and the shape and arrangement of rays in the male tail, including a ray 6 that lacks a tip opening to the outside and which tapers from a wide base [12].

Cultures were established by isolation of a single animal with a female soma. For gonochoristic species, we picked either a female with a copulatory plug, or one female and one male. The mode of reproduction was determined by isolating virgin L4 females/hermaphrodites and scoring for the presence of progeny. For selfing isolates, isogenic strains were produced by isolating a single larva for a few (3-6) generations. For male-female isolates, strains were established as isofemale lines or cryopreserved as large, multi-founder populations to maintain the sample's genetic diversity. Most strains are listed in WormBase (http://www.wormbase.org) and in http://www.justbio.com/worms/index.php and will soon be listed with distribution maps in RhabditinaDB (http://wormtails.bio.nyu.edu).

### Species identification and mating tests

Males and females from new *Caenorhabditis *isolates were compared at the morphological level with known species either by studying them alongside individuals from cultured strains or by consulting published species descriptions. If the morphology of a new isolate was not unique, mating tests were performed with individuals of morphologically similar strains.

#### Isolates with a male-female mode of reproduction

3-6 fourth larval stage (L4) females and 3-6 L4 or adult males were placed on a 55-mm Petri dish seeded with *E. coli *OP50 to mate. The cross was scored regularly for the presence of laid embryos, hatched larvae, and fertile adults. Most crosses were performed at least in duplicates. Many crosses between different *Caenorhabditis *species were successful at the mating behavior level, as evidenced by the presence of a mating plug deposited by the male on the female vulva, yet no embryos were laid. Other crosses produced dead embryos, and in some cases sterile female larval and adult progeny (Additional File 1). F_1 _sterility was assessed by placing F_1 _females either with sibling males from the same cross (if any) or males from either parental genotype.

#### Isolates with a selfing mode of reproduction

Hermaphrodites mostly produce hermaphrodite progeny upon selfing, and rare males by non-disjunction of the × chromosome. The cross-progeny of (cross-fertile) hermaphrodites and males consists of about 50% males. To test for cross-fertility, 3-6 hermaphrodites of one isolate were placed with 3-6 males of the other isolate on a 55-mm Petri dish seeded with *E. coli *OP50. The presence of numerous males (over 20%, much more than on control plates seeded with only hermaphrodites) on a plate indicates a successful cross and provides a test for biological species [19].

### PCR, sequencing and sequence alignment

We attempted to obtain partial sequences of 11 genes for all *Caenorhabditis *species: genes for SSU and LSU rRNA, the largest subunit of RNA polymerase II (RNAP2, *ama-1 *in *C*. *elegans*) as in [3] and *lin-44 *(encoding a Wnt signaling factor), *par-6 *(encoding a PDZ-domain-containing protein), *pkc-3 *(encoding an atypical protein kinase C) and the orthologs of *C. elegans *genes ZK686.3 (G43, orthologous to the putative tumor suppressor N33), W02B12.9 (G140, orthologous to the mitochondrial carrier protein MRS3/4), ZK795.3 (G3857, orthologous to a U3 small nucleolar ribonucleoprotein component), Y97E10AL.2 (OMCL4763, a predicted alpha/beta hydrolase) and Y45G12B.2a (OMCL4988, a predicted E3 ubiquitin ligase). Most of these genes were chosen for their expected information content for phylogenetic analysis as derived from genome sequences of six *Caenorhabditis *species (*C. elegans, C. brenneri, C. briggsae, C. remanei, C. japonica *and *C. angaria*) and some EST sequences for *C*. sp. 5 [20]. That is, from candidate genes with unambiguous alignments among 1:1 orthologs, we chose those which provided some resolution in a phylogenetic analysis with the above six species (data not shown). Such genes promised to have sufficient nucleotide variation to resolve relationships between closely related *Caenorhabditis *species. Degenerate primers were designed to regions conserved in these *Caenorhabditis *species and in *Pristionchus pacificus *(see Additional File 2). SSU and LSU rDNA was amplified from worm lysates as described previously [3]. Sequences of protein coding genes were amplified from cDNA as follows. Total RNA was isolated from mixed stage worms with the Qiagen RNeasy Mini kit following the tissue protocol or with Trizol. RT PCR was performed either with the Qiagen OneStep RT PCR kit using specific primers, or by first-strand cDNA synthesis (with the Transcriptor High Fidelity cDNA Synthesis kit (Roche) using anchored oligo-dT primers, or with the Protoscript kit by New England Biolabs, or with the Invitrogen Superscript III kit using random primers), followed by PCR with gene-specific primers. PCR products were purified and sequenced through Agencourt, or in-house with the Wizard VC Gel and PCR clean-up System (Promega) and the ABI BigDye Terminator v3.1 cycle sequencing kit (Applied Biosystems). Electrophoresis was performed on an ABI 3730 DNA Analyzer. Some PCR products were cleaned using the Zymoclean Gel DNA Recovery kit and sequenced at the University of Utah core facility. Sequences were assembled with Sequencher (Gene Codes) and deposited at GenBank under the accession numbers listed in Additional File 3. Alignments for protein coding genes were generated with ClustalX [21] and were in some cases manually improved by aligning the amino acid sequence. The rRNA sequences were also aligned with ClustalX. These alignments were largely unambiguous.

As barcodes for quick and easy identification of *Caenorhabditis *species, we explored the ITS regions between SSU rRNA and LSU rRNA. It proved easiest to amplify ITS1 and ITS2 separately with one primer in the highly conserved 5.8S region in each case (Additional File 2). Sequences were generated as described above. The sequences, which contained highly conserved anchor regions in the rRNA genes, were aligned using ClustalX or Muscle, which is optimized for aligning sequences with highly diverged segments, such as introns and intergenic sequences [22].

### Phylogenetic analysis

The data file used for phylogenetic analyses was a concatenated alignment of the eleven gene segments listed in the previous section (excluding ITS sequences, Additional File 4). Although some of these sequences were missing for some taxa, the full dataset was used for all phylogenetic analyses discussed here. There has been some controversy regarding the treatment of datasets with missing data in phylogenetic analyses [23-25]. Many simulations and tests with empirical data have demonstrated that using datasets in which some taxa are missing even large amounts of data do not generally suffer ill effects. Instead, better accuracy and resolution are generally obtained if characters with missing data are included than if they are excluded [25]. Consistent with these results, we also found that excluding characters with missing data resulted in poorer resolution (but not significantly different topologies) than if all characters were included (data not presented here). We thus only report our analyses with the full dataset.

To test the data for robustness to method of phylogenetic inference, we compared the results from analyses with weighted maximum parsimony (wMP), maximum likelihood (ML) and Bayesian inference (BI). Robustness of the data to character representation was tested using bootstrap and jackknife analyses.

The wMP analysis was performed with PAUP* ver4b10 [26]. A transversion was weighted twice a transition as in previous analyses of this taxon [3]. A jackknife analysis was performed with 1000 replicates and two addition sequence replicates in each round.

The ML analysis (a 100-replicate bootstrap and a thorough heuristic search) was run with RAxML ver. 7.2.8 ("BlackBox" version) via the CIPRES Science Gateway on the TeraGrid of NSF [27-30]. A six-parameter substitution model was used with a gamma correction for rate differences across sites (using 25 discrete categories of sites) and a correction for unvarying sites (GTR+Γ+I). Parameters were estimated from the data. The shape parameter for the gamma distribution of rates was α = 0.44081. Estimated proportions of nucleotides were: π(A) = 0.264, π(C) = 0.217, π(G) = 0.260, π(T) = 0.259. Estimated rates for the GTR model were: *f*(AC) = 1.390, *f*(AG) = 3.120, *f*(AT) = 1.182, *f*(CG) = 1.115, *f*(CT) = 5.790, relative to *f*(GT) = 1.000.

Another analysis of the same dataset was performed using Bayesian Inference (BI) as implemented in MrBayes ver. 3.1.2 [31,32] via the CIPRES portal [27,28,33]. A six-parameter substitution model was used with a gamma correction for rate differences across sites and an estimate for the proportion of invariant sites (GTR+Γ+I). The analysis was stopped automatically by MrBayes at 4,055,000 generations (due to convergence of all parameters). Trees and parameters were sampled every 1000 generations for a total of 1,556 samples. Burnin was set to 50% of the samples to calculate the clade credibility values (posterior probabilities) and to estimate the model parameters, which were: π(A) = 0.265, π(C) = 0.217, π(G) = 0.256, π(T) = 0.262. Estimated rates for the GTR model were: *f*(AC) = 1.296, *f*(AG) = 2.284, *f*(AT) = 1.108, *f*(CG) = 1.147, *f*(CT) = 3.678, relative to *f*(GT) = 1.000. In the final tree, only one branch had a clade credibility value less than 100 (i.e., a branch that placed *C*. sp. 20 with *C. angaria*, *C. drosophilae, C*, sp. 2, 8 and 12, exclusive of *C*. sp. 6 and 13).

### Genetic divergence

To estimate the genetic divergence within *Caenorhabditis *(and for comparisons with other taxa), we calculated the amount of nucleotide change along the branches of the phylogeny using maximum likelihood implemented in PAUP* and the sequences of RNAP2. This gene was used because previous results showed that the rRNA genes--but not RNAP2--display significant heterotachy [3], and RNAP2 was the only protein-coding gene that we could sequence for all species. All parameters for a general time-reversible model were estimated from the data. For comparison, we also calculated branch lengths for an RNAP2 dataset from 12 *Drosophila *species with the topology from [14]. The data matrix is found in Additional File 5.

### ITS2 Barcodes

To test the utility of ITS2 sequences (i.e. the intergenic region between 5.8S and LSU rRNA genes) for distinguishing which isolates belong to which *Caenorhabditis *species, we sequenced this region for several strains of the species that were isolated more than once. The sequences, which contained the highly conserved 5.8S rDNA at the 5' end and part of the LSU rDNA at the 3' end, were aligned with ClustalX and then trimmed down to the ITS2 sequence only, following the annotation of the rRNA gene structure of *C. elegans *[34]). This data matrix is presented in Additional File 6. This alignment was used to determine the pairwise differences between species and strains. To represent these differences graphically, we calculated the branch length of a tree for all strains. This tree was reconstructed based on the ITS2 sequences with MP, using the species phylogeny as a constraint. A heuristic search yielded eight most parsimonious trees, one of which was chosen for further analysis. Branch lengths of this tree were determined by parsimony and include indels (one change per gap, regardless of the length of the gap). Changes with ambiguous branch assignment were optimized with ACCTRAN. ACCTRAN was used to offset somewhat the underestimation by parsimony of changes occurring in deeper branches. Correcting for superimposed changes demonstrates even greater discernibility between intra- and interspecific differences, suggesting that the parsimony approach is conservative. Pairwise intraspecific differences were manually tabulated as transitions, transversions, and indels.

## Results and discussion

### Sampling of rotting plant parts yielded many *Caenorhabditis *isolates including 16 new species

**Figure 1:**
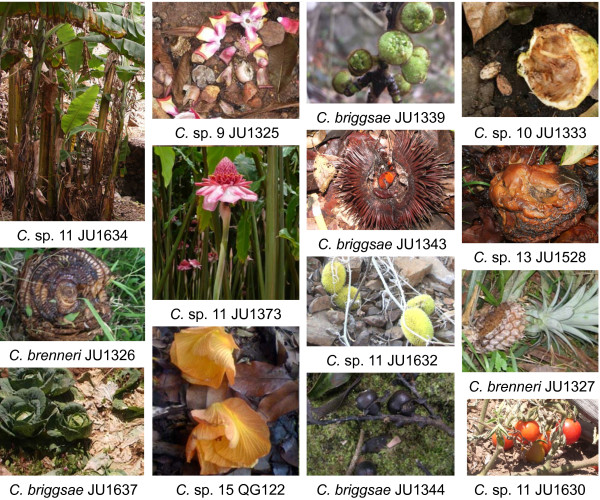
**Rotting substrates from which *Caenorhabditis *species were isolated**. Examples of sampled plant parts from which *Caenorhabditis *isolates could be successfully isolated. Most pictures illustrate the rotting sample on the ground, while a few others show the corresponding non-rotten plant in the same location. See additional files 7 and 8 for identifications and further sampling. First column: banana pseudo-stems, cabbage leaves. Second column: rotting flowers (mixed flowers, torch ginger, *Hibiscus *flower). Third column: rotting wild fruits (figs, chestnut, cucumber *(Cucumis)*, unidentified). Fourth column: rotting cultivated fruits (cocoa, apple, pineapple, tomato). Pictures: MAF, except the *Hibiscus *flower (MVR) and the torch ginger flower (yielding JU1373), courtesy of V. Robert.

We systematically sampled rotting fruits and found that roughly one third of the samples contained at least one *Caenorhabditis *species. In rotting fruits, *Caenorhabditis *nematodes were present as adults and larvae of all stages, sometimes in large numbers. This is in contrast to previous records from soil and even from compost, where most individuals were in the dauer larva stage [35]. We further sampled other types of rotting vegetal material (Figure 1) and failed to find *Caenorhabditis *species in wood. However, we did find them in rotting flowers, plant stems, and sometimes in leaves (Additional Files 7 and 8).

By sampling rotting fruits, flowers and stems in various temperate and tropical regions of the world, several hundred cultures of different *Caenorhabditis *species were established. Crosses with established cultures of known species revealed that many of the new isolates belonged to four already described species, namely *C. elegans*, *C. briggsae*, *C. remanei *and *C. brenneri *(Additional File 7).

Sixteen additional species were identified by unique morphology or by the absence of fertile cross-progeny when mated with isolates of previously known species with similar morphology (Additional File 1). Comparisons with published species descriptions suggest that all of these species are new to science. We provisionally refer to these newly cultured species by numbers, sp. 5 to sp. 20 (Table 1).

**Table 1:** New *Caenorhabditis *species, region where they were isolated and reproductive mode

Species number	Representative isolate(s)	Geographic location(s)	Mode of reproduction
sp. 5	JU727	China	gonochoristic (male-female)

sp. 6	EG4788	Portugal	gonochoristic

sp. 7	JU1199	West Africa	gonochoristic

sp. 8	QX1182	Eastern USA	gonochoristic

sp. 9	JU1325 EG5268	South India Congo	gonochoristic

sp. 10	JU1333	South India	gonochoristic

sp. 11	JU1373	La Réunion	hermaphroditic
	JU1428	French Guiana	

	JU1630	Cape Verde	

	EG5889	Puerto Rico	

	JU1975	Brazil	

	QG131	Hawaii	

sp. 12	JU1426	French Guiana	gonochoristic

sp. 13	JU1528	France	gonochoristic

sp. 14	EG5716	Moorea	gonochoristic
	JU1905	Guadeloupe	

sp. 15	QG122	Hawaii	gonochoristic

sp. 16	JU1873	Indonesia	gonochoristic

sp. 17	JU1825	French Guiana	gonochoristic

sp. 18	JU1857	French Guiana	gonochoristic

sp. 19	EG6142	Puerto Rico	gonochoristic

sp. 20	NIC113	Guadeloupe	gonochoristic

Some of the new species were sampled many times, whereas some others are currently represented by single isolates (Additional File 8). For the species sampled many times, we could not find any clear substrate preference. For example, *C*. sp. 11 was found in rotting flowers, fruits and banana pseudo-stems, like *C. briggsae*. All of these habitats, however, are rich in nutrients, bacteria and likely yeasts, and may provide similar conditions as habitats for the species.

### Phylogenetic relationships

**Figure 2:**
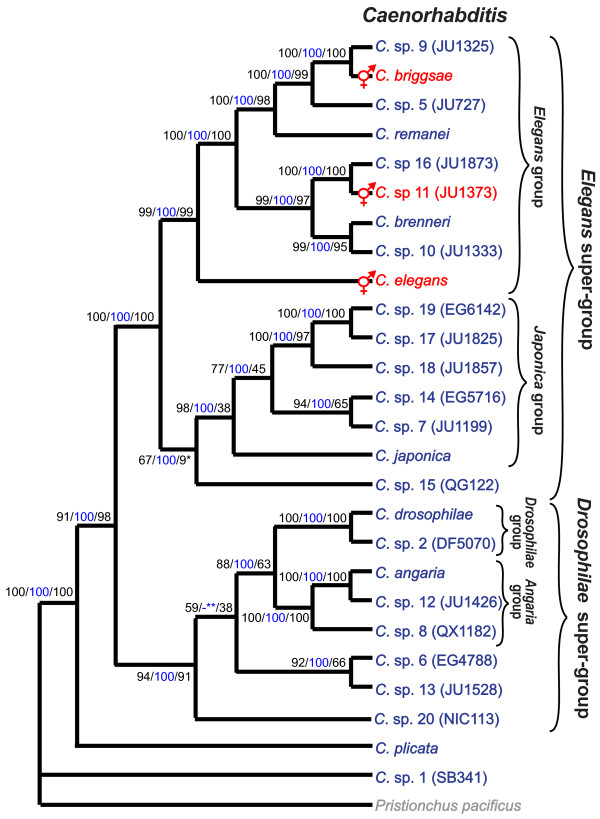
**Our current best hypothesis for the phylogenetic relationships of all *Caenorhabditis *in culture and convergent evolution of hermaphroditism**. Depicted is result of the maximum likelihood bootstrap analysis. Numbers on branches show, respectively, the support values for 100 bootstrap repeats in the ML analysis in percent, the posterior probabilities from the Bayesian inference analysis (blue), and the support values for 2000 jackknife repeats in a weighted maximum parsimony analysis in percent. * Weighted maximum parsimony analysis favors a position of *C*. sp. 15 as the sister species of the *Elegans *group with 60% support. ** Bayesian inference favors *C*. sp. 20 to form the sister species of the *Angaria *and *Drosophilae *groups with a clade credibility value of 96, the lowest in this analysis. Three species (in red) reproduce as self-fertilizing hermaphrodites with rare males, whereas all other species (in blue) are gonochoristic with females and males at approximately equal proportions. Hermaphroditism evolved convergently in all three lineages.

To elucidate the phylogenetic relationships of the 26 *Caenorhabditis *species in culture, we performed phylogenetic analyses with three methods, maximum likelihood bootstrap (ML) using RAxML, Bayesian inference (BI) using MrBayes, and weighted maximum parsimony (wMP) using PAUP*. All methods resulted largely in the same topology with high support for most branches in the ML and BI analyses (Figure 2). There is some uncertainty about the positions of *C*. sp. 15 and *C*. sp. 20 which differ from those shown in Figure 2 in the wMP analysis (*C*. sp. 15) or in the BI analysis (*C*. sp. 20), respectively. However, the support for the alternative placements of these species is low in each case.

The phylogeny supports the following grouping: *C*. sp. 1 branches off first and the second branch is *C. plicata*. This branching pattern is the same as in a previous analysis with only 11 *Caenorhabditis *species but with 54 species outside of *Caenorhabditis *[2], suggesting that the choice of *P. pacificus *as the outgroup representative had no effect on the overall tree topology. The remaining *Caenorhabditis *species fall into two monophyletic groups, the *Elegans *super-group and the *Drosophilae *super-group (Figure 2). Within the *Elegans *super-group, we find two subclades, which we call the *Japonica *group and the *Elegans *group. The *Japonica *group consists of *C*. spp. 7, 14, 17-19 and *C. japonica*. The monophyly of this group is well supported by likelihood analyses and less so by wMP. *C*. sp. 15 appears to be the sister species of the *Japonica *group. The *Elegans *group comprises the remaining *Elegans *super-group species. Their relationships are highly supported in all analyses. *C. elegans *forms the first branch of this group. The other *Elegans *group species fall in two clades, one comprising *C. briggsae, C. remanei *and *C*. spp. 5 and 9, the other one comprising *C. brenneri *and *C*. spp. 10, 11 and 16. None of the 26 *Caenorhabditis *species in this analysis is the sister species of *C. elegans*. Within the *Drosophilae *super-group, *C. drosophilae *and *C*. sp. 2 form the *Drosophilae *group which is the sister taxon to the highly supported *Angaria *group composed of *C*. *angaria *plus *C*. sp. 12 and *C*. sp. 8. Of the other three species in the *Drosophilae *super-group, *C*. sp. 6 and 13 are sister species, and *C*. sp. 20 possibly forms the first branch.

### Genetic divergence

**Figure 3:**
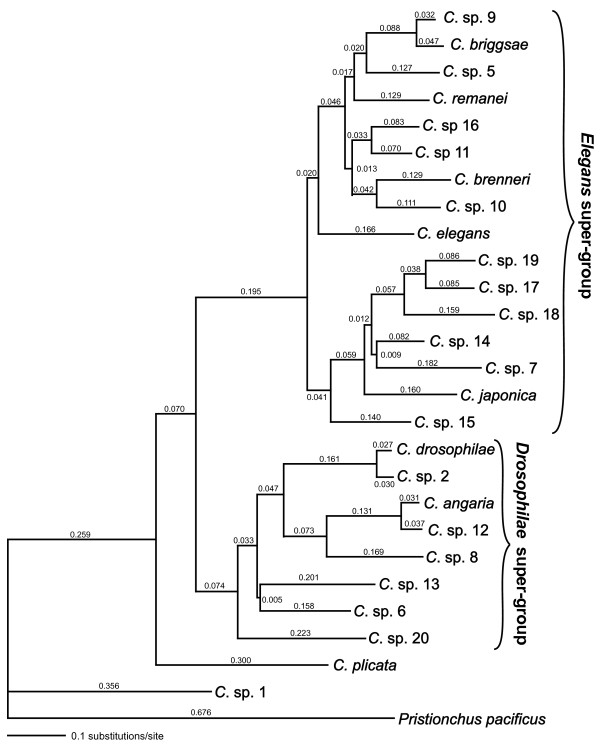
**Likelihood phylogram for RNA polymerase II genes**. The species relationships follow the phylogeny in Fig. 2. A general time-reversible model was used to estimate branch lengths (GTR+Γ+I, parameters estimated from the data).

The genetic divergences of RNAP2 between the *Caenorhabditis *species are depicted as the lengths of the branches of the phylogram in Figure 3. With few exceptions, the branches leading to individual species are longer than the internal branches. The two longest internal branches are those leading to the *Elegans *super-group and the branch separating *C*. sp. 1 from the rest of *Caenorhabditis*. The smallest genetic divergence between two species is found between *C. briggsae *and *C*. sp. 9 (0.079 substitutions/site), between *C. angaria *and *C*. sp. 12 (0.068 substitutions/site), and between *C. drosophilae *and *C*. sp. 2 (0.057 substitutions/site). Correspondingly, the first two species pairs produce viable hybrids in mating experiments. The divergence between these species pairs is comparable to that between *Drosophila yakuba *and *D. erecta *(0.048 substitutions/site in RNAP2, see Additional File 9). For RNAP2, the genetic divergence spanned by the *Elegans *group (*C. briggsae-C*. sp. 18: 0.566 substitutions/site) is similar to that spanned by the *Sophophora *subgenus of *Drosophila *(*D. melanogaster-D. willistoni*: 0.621 substitutions/site). Thus, we now have a similar range of genetic divergence represented in the *Caenorhabditis *species as for *Drosophila *species. One caveat remains: we still do not know if there exists a species which is more closely related to *C. elegans *than *C. briggsae *or any other individual species, and the relatively long branch to *C. elegans *remains "unbroken".

### World distribution

**Figure 4:**
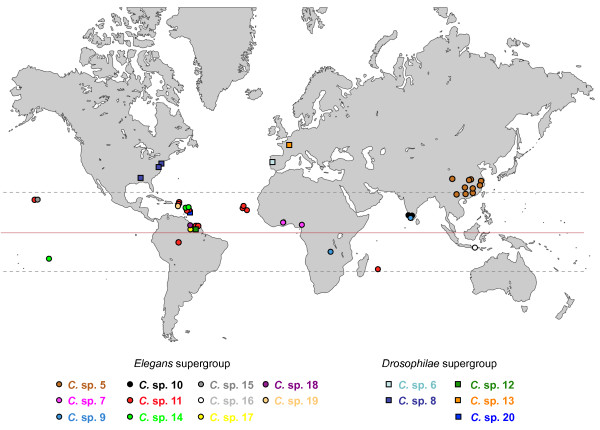
**World distribution of new *Caenorhabditis *species discovered since 2005**. Based on the strains listed in Additional File 8 and [54]. Squares: *Drosophilae *super-group species. Circles: *Elegans *super-group species.

The four described species of the *Elegans *group occur on several continents. The restriction of the distribution of *C. remanei *to temperate regions and of *C. brenneri *to tropical regions reported previously [36] is further corroborated by our findings. The geographic distribution of the new *Caenorhabditis *species is depicted in Figure 4. Of the new species, only *C*. sp. 6, 8 and 13 were found in temperate regions and *C*. sp. 5 was found in temperate and tropical regions. All remaining new species were sampled from tropical sites. Some of the species that have been sampled several times are shared between different tropical regions. *C*. sp. 11 was found in La Réunion in the Indian Ocean, Puerto Rico and the Cape Verde Islands in the Atlantic Ocean, French Guiana and Brazil in South America and Hawaii in the Pacific Ocean. *C*. sp. 9 has been found in Central Africa and South India. By contrast, *C*. sp. 5 has remarkably been found exclusively in East Asia (China and Vietnam), *C*. sp. 7 in West Africa (Ghana, Nigeria), *C*. sp. 8 in the Eastern half of United States and *C*. sp. 10 in South India. At a supraspecific level, the trio of *C*. spp. 17, 18 and 19 was found in the Neotropics, while the noncosmopolitan species of the sister group of *C. elegans *were all collected in Asia. *C*. sp. 20 and the species of the *Angaria *group and *C. drosophilae *were found in the tropical and temperate Americas. *C*. sp. 2 was isolated from islands in the Mediterranean and the Eastern Atlantic Ocean, however, its association with cacti and cactophilic flies indicates that *C*. sp. 2 is also an American species which we currently only know from locations to which it has been introduced in historical times [11]. Thus, with the exception of the two European species *C*. sp. 6 and 13, all members of the *Drosophilae *super-group are New World species.

The temperature preferences of the species correlate with the latitude of their geographic distribution. Indeed, different from *C. elegans*, *C. briggsae *and *C. remanei*, the tropical species *C*. spp. 7, 9, 10, 11 do not grow at 15°C. In contrast, *C*. spp. 9, 10 and 11 can grow at 30°C (but not 33°C), a character they share with *C. briggsae *but not with *C. elegans *[[37]; M. Ailion, unpublished observations].

Several species often co-occur in the same location and sometimes in the same individual fruit. For example, *C. briggsae*, *C. brenneri *and *C*. sp. 10 were all found in the same garden in Kanjirapally, Kerala, India; *C. elegans*, *C. briggsae *and *C*. sp. 13 co-occurred in rotting apples in an orchard in Orsay, France; *C. briggsae*, *C. brenneri *and *C*. sp. 11 were found together in one garden in La Réunion; *C. briggsae *and *C*. sp. 14 were isolated from the same chestnut in Moorea; *C. elegans *and *C*. sp. 6 were found in rotting apples from the same tree in Amares, Portugal; *C. briggsae *and *C*. sp. 8 were found in the same rotting persimmon in New York; and *C. briggsae *and *C*. sp. 15 were found in the same small sample of rotting flowers in Kauai, Hawaii.

### Character evolution

To determine whether any evolutionary pattern for phenotypic characters can be discerned, we mapped several such characters onto our phylogeny (Additional File 10). Most informative morphological characters in rhabditids are associated with the male reproductive organs [12,38]. We therefore analyzed the 26 species for the shapes of their spicules and of their male tail with its fan and sensory rays. The evolution of reproductive modes is also of particular interest in *Caenorhabditis*.

#### Reproductive mode

*Caenorhabditis *species have one of two modes of reproduction. They can be gonochoristic (male-female), like *C. remanei *and *C. brenneri*, or they can be androdioecious with selfing hermaphrodites and facultative males, like *C. elegans *and *C. briggsae*. Of the new species, only *C*. sp. 11 presents the selfing mode of reproduction (Table 1). Tracing this character along the branches of the phylogenetic tree of *Caenorhabditis *reveals that hermaphroditism likely evolved independently in each of the three lineages (Figure 2). Assuming that evolution of hermaphroditism and gonochorism are equally likely, this scenario requires three evolutionary steps, whereas the alternative hypothesis that hermaphroditism evolved once requires six evolutionary steps (one gain of hermaphroditism and five reversals to gonochorism). Recent studies [39-42] have discovered multiple differences in the genetic underpinnings of sex determination in *C. elegans *and *C. briggsae*, supporting the hypothesis that hermaphroditism evolved convergently in these two species.

#### Morphology and other phenotypic characters

**Figure 5:**
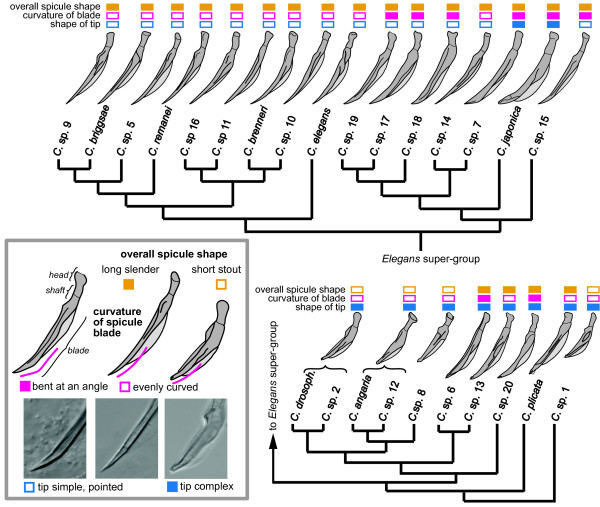
**Evolution of spicule shape**. For each *Caenorhabditis *species, a drawing of the spicule is shown in right lateral view with the dorsal side to the right (the spicules of *C. drosophilae *and *C*. sp. 2 and of *C. angaria *and *C*. sp. 12 are identical and are shown for only one species). Three features of the spicule are distinguished, each with two alternative character states: the overall shape (orange boxes), the curvature of the spicule blade (pink boxes) and the shape of the spicule tip (blue boxes). The character states for each species are indicated by filled or empty colored boxes above the images.

The spicules are the male copulatory organs, paired cuticular structures which generally consist of a head, a shaft and a blade which tapers to a tip. In all *Caenorhabditis *species, each spicule has a thin velum at the dorsal side and a seam which separates the spicule head from the spicule blade near the middle of the shaft (Figure 5). Differences are found in the shape of the distal part of the spicule blade which can be broad and bent at an angle or more evenly curved. The spicule tip can be complex with notches or small cuticular wings, or simple, tapering down to a narrow point. The general shape of the spicule can be short and stout, as in *C*. sp. 1 and *C*. *angaria*, or longer and narrower. The distribution of these characters on the tree reveals conflicts: all species outside of the *Elegans *super-group have a non-pointy, complex spicule tip, but so do *C. japonica *and *C*. sp. 7. The most parsimonious scenario for the evolution of this character is that the simple spicule tip, as it is present e.g. in *C. elegans*, evolved in the stem species of the *Elegans *super-group and was reversed to a more complex tip twice independently in *C. japonica *and *C*. sp. 7. The slender, evenly curved shape of the spicule is found in all species of the *Elegans *group and in *C*. sp. 14. Another character with a similar distribution is the frequency of division of the anterior vulval precursor cell P3.p, which is lower in species of the *Elegans *group and in *C*. sp. 14 [43]. These characters would be nonhomoplasiously distributed if *C*. sp. 14 were part of the *Elegans *group or its sister species, but our analyses place *C*. sp. 14 in the middle of the *Japonica *group with good support. Shorter and stouter spicules are found in *C*. sp. 1 and in the outgroup (i.e. *Protorhabditis *species [2]) as well as in the *Drosophilae *and *Angaria *groups. This distribution suggests that the spicule length increased after *C*. sp. 1 branched off and was then reduced again in the stem species of the clade consisting of the *Drosophilae *and *Angaria *groups.

**Figure 6:**
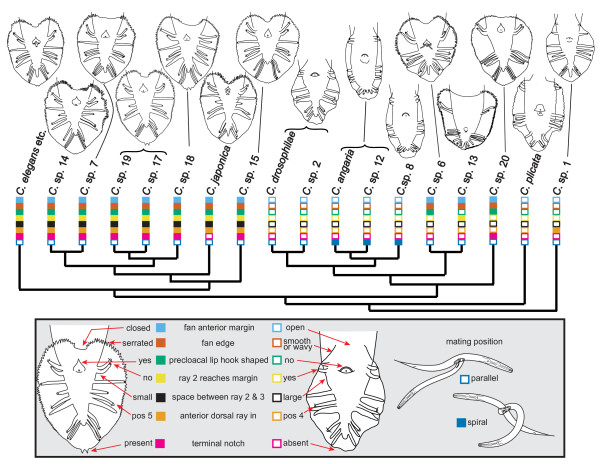
**Evolution of male tail characters**. Drawings of the male tail in ventral view are shown above the *Caenorhabditis *phylogeny. The male tail possesses a cuticular fan around the cloaca. Nine pairs of sensory rays are embedded in the fan. Differences between species are found in the shape of the anterior margin and the terminal end of the fan, in the arrangement of the rays and in the shape of the precloacal lip (cf. [12]). Seven characters of the male tail with two character states each are mapped onto the tree. The mating position is included as an eighth character. The spiral mating position is found only in the *Angaria *group (*C. angaria*, *C*. sp. 12 and *C*. sp. 8). It is correlated with a particularly narrow fan (compare the images). Male tails are largely identical in all species of the *Elegans *group, in *C. drosophilae *and *C*. sp. 2 and in *C. angaria *and *C*. sp. 12.

Conflicts also exist in the distribution of characters of the fan, a lateral extension of the cuticle at the male tail which is used during copulation (Figure 6). The fan contains nine pairs of sensory rays and the opening of the chemosensory phasmids. In all species of the *Elegans *super-group, the fan is heart-shaped and anteriorly closed and its edge is serrated. These features are derived within *Caenorhabditis*. *C*. sp. 1, *C. plicata *and the most closely related species of the outgroup, *Protorhabditis *[2] (see [38] for the characters of the *Protorhabditis *stem species), have oval open fans with a smooth or wavy edge. However, within the *Drosophilae *super-group, both types of fans occur. The fans in *C*. sp. 6 and *C*. sp. 20 have the typical heart shape. The fan in *C*. sp. 13 is unique in its squarish shape and unusual arrangements of rays, but it is anteriorly closed and has a serrated edge. The other species have an open and narrow fan, especially *C. angaria, C*. spp. 8 and 12. The distribution of these characters on the phylogeny suggests that an anteriorly closed, heart-shaped fan with a serrated edge evolved in the stem species of the *Elegans *and *Drosophilae *super-groups and was reversed to a relatively narrow, open fan with a smooth edge in the stem species of the *Drosophilae *and *Angaria *groups. A heart-shaped fan always co-occurs with a hook-shaped precloacal lip. Thus, the same evolutionary scenario can be assumed for this character. Interestingly, in *C*. sp. 13, both the shape of the fan and the precloacal lip have been modified together as well (this species has a squarish fan and lacks the hook).

Another conflict between the molecular data (supporting the phylogeny presented here) and a morphological character concerns the position of one of the three pairs of rays which are attached to the dorsal surface of the fan. In all species of the *Drosophilae *super-group, the three dorsal rays are in positions 1, 4 and 7, counted from anterior, whereas in all species of the *Elegans *super-group the dorsal rays are in position 1, 5 and 7 (Figure 6). Thus, the middle dorsal ray is in a different position. A middle dorsal ray in position 5 is also found in *C*. sp. 1 and in the *Caenorhabditis *sister group (the *Protorhabditis *group, see [38] for characters of the stem species), but not in *C. plicata*. Since the position of *C*. sp. 1, *C. plicata*, the *Drosophilae *super-group and the *Elegans *super-group are well supported, the distribution of this character requires at least two evolutionary steps, either two shifts of the middle dorsal ray from position 5 to 4 in the *Drosophilae *super-group and in *C. plicata*, or one shift from position 5 to 4 after the branch to *C*. sp. 1 and a reversal to the ancestral situation (middle dorsal ray in pos. 5) in the *Elegans *super-group. Likewise, the occurrence of a particularly short ray 4 is homoplasious, as it is found in *C. japonica, C*. sp. 14, 17 and 19. The distribution of this character requires at least three evolutionary steps.

The ability to start an RNA interference response upon external administration of double-stranded actin RNAs was tested on all new species (Nuez and Félix, submitted). This character has a complex distribution. Competence to external RNAi is absent in all species of the sister clade of *C. elegans*, in the clade comprising *C*. spp. 17, 18 and 19, in *C. plicata *and in all species of the *Angaria *and *Drosophilae *group but not in *C*. sp. 6, *C*. sp. 13 and *C*. sp. 20 (Nuez and Félix, submitted). Mapping of this character onto the phylogeny suggests that competence to respond to external dsRNA may have been present in the *Caenorhabditis *stem species and was lost four times independently.

There are also characters which are mostly unambiguously distributed and thus constitute reliable apomorphic characters for certain monophyletic groups that are supported by molecular data: In most species of the *Elegans *super-group, the distal end of the fan is notched. This terminal notch is particularly large in the monophyletic group consisting of *C*. sp. 17, 18 and 19. The *Elegans *super-group is characterized by a small space between the second and third ray (smaller than the space between the third and fourth ray or of similar size), when compared to the other *Caenorhabditis *species in which this space is always bigger (Figure 6). A particularly narrow fan is found in *C. angaria, C*. sp. 8 and *C*. sp. 12. Also, these species share papilliform phasmids, a very similar spicule shape, and a spiral mating position in which the male is coiled around the female (Figures 5 and 6). In addition, *C. angaria, C*. sp. 8 and *C*. sp. 12 all have a short stoma with a bifurcated projection at each sector of the metastegostom (see illustrations in [44] for *C. angaria)*. Thus, the monophyly of the *Angaria *group is exceptionally well supported by morphological and molecular characters. A number of previously mentioned phenotypic characters support the sister group relationships of the *Angaria *and *Drosophilae *groups even though their distribution across all *Caenorhabditis *species is homoplastic: In all species of both clades the spicule is short, the fan is open and lacks a serrated edge, and all species are insensitive to externally administered double-stranded RNA (against actin). A sister group relationship of these two clades has been proposed earlier [44], based on molecular data and on the presence of one semicircular flap on each lip seen on SEM images of *C. angaria *and *C. drosophilae*. The presence of this flap needs to be confirmed for the other species of these groups, but could constitute a further apomorphy. The geographic distribution of their members suggest that the *Angaria *and *Drosophilae *groups originated in the New World.

### ITS2 is a suitable barcode for distinguishing *Caenorhabditis *species

Morphology can be used to assign species to the major groups within *Caenorhabditis*, but some species within these groups look very similar or entirely alike. In fact, the genus contains a host of morphological sibling species. Therefore, morphology alone is not suitable for identifying new species. In this study, species were initially identified via mating tests (Additional File 1). However, with a growing number of species, mating tests become tedious and time-consuming. Unlike morphology, genomic sequences contain many easily accessible species-specific differences. We thus sought a genetic barcode for *Caenorhabditis *species.

As suitable targets for barcoding in nematodes, De Ley et al. [45] proposed the use of SSU and LSU rDNA sequences. However, within *Caenorhabditis*, SSU rDNA is very highly conserved and can be identical in closely related species (e.g. in *C*. *angaria *and *C*. sp. 12). LSU rDNA, specifically the D2D3 region, is usually more variable, but *C. briggsae *and *C*. sp. 9 differ in only five positions over the entire LSU locus (three substitutions and two indels). Such a small number of differences can be easily concealed by sequencing errors. Here, we explore the ITS region instead. Both internal transcribed spacers are variable between species. In more distantly related species, the unambiguously alignable portions of the ITS regions are very short. However, the flanking rRNA gene sequences are highly conserved. Thus, PCR with primers in the flanking sequences reliably amplifies the ITS regions. The flanking sequences can also serve as anchors for alignments.

The ITS1 region was often variable within a species and even within one animal, making direct sequencing of PCR products problematic (data not shown). ITS2 was less polymorphic in the strains tested, although here, too, we found four strains with indel polymorphisms that precluded sequencing of the entire region without cloning. Nevertheless, the parts of ITS2 that could be sequenced directly from PCR products were long enough to enable species identification.

**Figure 7:**
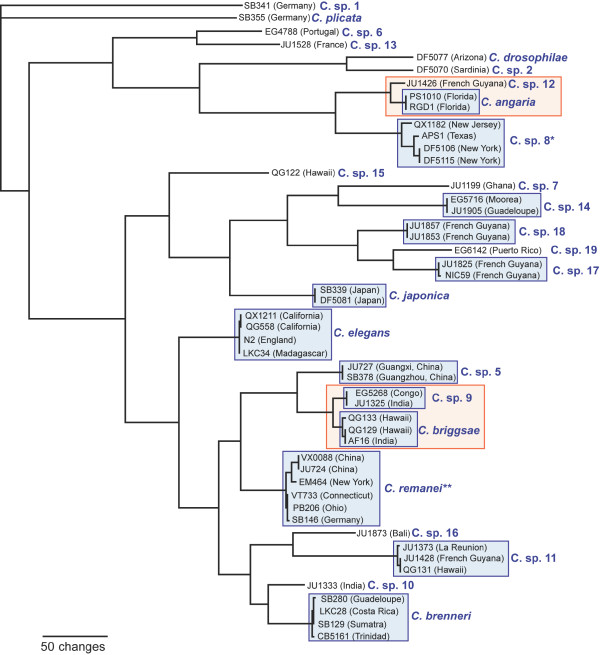
**Graphic representation of differences in the ITS2 region between and within *Caenorhabditis *species as branch lengths of a tree for 50 *Caenorhabditis *strains**. Branch length was determined by maximum parsimony (see Methods). With one exception (* *C*. sp. 8), the differences between strains of the same species (blue boxes) are smaller than the smallest differences between the two most closely related species pairs (orange boxes). In all cases, the differences separating any pair of species is much greater than the differences separating strains of the corresponding species. **The differences between *C. remanei *strains are larger than the differences seen within other species. Recently, however, hybrid breakdown has been observed in matings between strain VX0088 from China and several strains from Ohio, congruent with the long ITS2 branch of the Chinese isolates (Asher Cutter and Alivia Dey, pers. comm).

To see whether the genetic divergence between different isolates of the same biological species (as determined by mating tests) was smaller than that between species, we sequenced the ITS2 region of at least two strains from several species which have been isolated more than once. We found that, with one exception, the differences between two strains were smaller than the differences between two most closely related species pairs, *C. briggsae *and *C*. sp. 9 (AF16 vs. JU1325: 20 substitutions and six indels) and *C*. *angaria *and *C*. sp. 12 (24 substitutions, 7 indels). (Figure 7 and Additional File 11). Aside from *C*. sp. 8 (see below), the most differences were found between *C. remanei *strains with 11 substitutions and 1 indel between the two most dissimilar strains (e.g. VX0088 from China and PB206 from Ohio). Recent results of mating tests between strains from China and Ohio assigned to *C. remanei *showed hybrid breakdown in the F_2 _generation, indicating that these strains may actually belong to two separate biological species (Asher Cutter and Alivia Dey, pers. comm.). The strain EM464 from New York also differs by ten substitutions from other *C. remanei *strains from Ohio and Germany and it remains to be tested whether hybrids between these strains are fully fertile. To our surprise, we found large differences between the strains of *C*. sp. 8, all collected from locations in the Eastern USA. The ITS2 sequence of these strains has an area with imperfect repeats which in strain QX1182 is 199 nucleotides shorter than in strain DF5106 (50 vs. 249). The same region contains indel polymorphisms in strain APS1 and could not be sequenced from PCR products. Despite these differences, *C*. sp. 8 can be easily identified based on the ITS2 region. Thus, this molecule serves as a reliable and convenient barcode for distinguishing samples of different *Caenorhabditis *species.

## Conclusions

### *Caenorhabditis *are "fruit worms", not soil nematodes

Our study has shown beyond doubt that the best place to find *Caenorhabditis *species is rotten fruit. Of the 26 *Caenorhabditis *species currently in culture, all but seven have been isolated from rotten fruit, although most species have also been found in other rotting plant material. We do not yet know whether any *Caenorhabditis *species is a strict fruit specialist, but it is likely that *C. japonica *is specific for the fruit of *Schoepfia jasminodera *[46]. The information which we have gathered to date shows that the habitats of these nematodes are strikingly similar to those of *Drosophila *species. Many *Drosophila *species, including *D. melanogaster*, can be found in various fermenting fruit and fungi, but there are also specialists for particular fruit like figs or those that specialize on breeding in flowers [47]. Furthermore, some *Drosophila *species are specialists for rotten cactus, as are *C. drosophilae *and *C*. sp. 2 [11], and others breed in fungi, a habitat from which *C*. sp. 1 and *C. auriculariae *have been isolated [11]. Further samplings of *Caenorhabditis *species may discover more parallels between the habitats of these genera. It should be noted that at least two *Caenorhabditis *species live in a habitat quite different from rotten fruit, namely decomposing animal tissue. *C. plicata *has only been found in carrion and *C. bovis *(not included here for the lack of material for molecular analyses) lives in the inflamed ears of cattle [11]. Taking all of our sampling data together, it is clear that *Caenorhabditis *are not soil nematodes. The only stage which is occasionally found in soil is the dauer larva. In this respect, *Caenorhabditis *species do not differ much from the majority of rhabditid nematode species which also reproduce in substrates rich in nutrients and bacteria. Nematodes that specialize in such habitats often use other animals for dispersal. Such phoresy is indeed an essential part of the life cycle of *C. angaria, C. drosophilae *and *C. japonica *where the dauer larvae attach to weevils, drosophilid flies or burrower bugs, respectively [44,46,48]. Other species, including *C. elegans *and *C. briggsae*, have been isolated from phoretic carriers [11] and it is likely that phoretic relationships exist for many or all other *Caenorhabditis *species.

### How many *Caenorhabditis *species are there?

**Figure 8:**
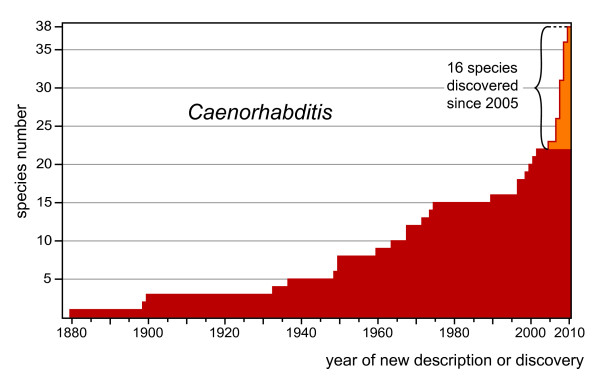
**Discovery rate of *Caenorhabditis *species**. The number of *Caenorhabditis *species is plotted cumulatively by year of description or discovery (if known). As of 2010, there were 38 *Caenorhabditis *species; this is a maximum number, since 6 of the 20 described species are not very well known and are potentially synonymous with other species [11]. The 16 species reported in this study were discovered between 2005 and 2010. The rate of discovery has increased since sampling efforts have focused on rotting fruit and other decaying plant material. The shape of this curve suggests that only a fraction of *Caenorhabditis *species is presently known.

Extensive collecting of *Caenorhabditis *from rotting plant material has yielded new species even from regions that were already fairly well sampled, e.g. Europe and the Eastern United States (*C*. spp. 6, 8 and 13). Over the last six years, 16 new species were found (Figure 8). We therefore expect that the discovery of new *Caenorhabditis *species will continue. It is likely that we have only scratched the surface of *Caenorhabditis *biodiversity.

### Remarks on the geographic range

Our new records corroborate the observation that of all *Caenorhabditis *species, only *C. briggsae *and *C. elegans *are cosmopolitan (Figure 4 and Additional File 12). However, only *C. briggsae *seems to be equally common in temperate and tropical regions. *C. elegans *was recorded from tropical Africa at high altitude sites (2000 m and above) in Limuru, Kenya [49] and Addis Ababa, Ethiopia (data courtesy of Dee Denver) and on islands such as La Réunion (at altitude 1100 m) or Hawaii (at an unknown altitude). Two other species have been found in temperate as well as in tropical regions: *C. plicata *was found in Germany and Kenya and *C*. sp. 5 occurs in temperate and tropical China and in Vietnam. The remaining *Caenorhabditis *species for which we have more than one record were either isolated from tropical or from temperate regions but not from both. Of the 16 *Elegans *super-group species in culture, ten were found exclusively in the tropics and only two (*C. remanei *and *C. japonica*) are exclusively temperate. In contrast, only three of the ten non-*Elegans *super-group species are only found in the tropics. This suggests a radiation of *Elegans *super-group species occurred in tropical regions. However, this picture can easily change in the future, since our knowledge of *Caenorhabditis *biogeography is still very sketchy. The southern hemisphere has been only sparsely sampled and there is almost no information about the distribution of *Caenorhabditis *species in southern temperate regions.

### Phylogeny and character evolution

In this study, we used the sequences of 11 genes to reconstruct the phylogeny of *Caenorhabditis *species. Our analyses yielded a tree with some very well-resolved areas, but there are also branches which are weakly supported. More sequence information is needed to resolve all branches with high confidence. However, the current phylogeny is a usable working hypothesis which allows us to draw some sound conclusions: (1) *Caenorhabditis *comprises two monophyletic sister groups, the *Elegans *super-group with 16 species currently in culture, and the *Drosophilae *super-group with eight species. (2) None of the species in the *Elegans *super-group is more closely related to *C. elegans *than *C. brenneri*, *C. briggsae *or *C. remanei*; i.e., we do not yet know of an extant sister species of *C. elegans*. (3) Morphologically, the *Elegans *super-group species are more uniform than the *Drosophilae *super-group species, making it particularly difficult to identify any of the individual *Elegans *super-group species by morphology alone. (4) With the exception of *C. briggsae *and *C*. sp. 9, all of these species are fully reproductively isolated and genetically divergent.

A closer look at a small number of morphological characters showed that almost none of them had an unambiguous distribution on our current phylogeny. For almost all morphological characters, conflicts existed between each other and/or the molecular data that were used to reconstruct the phylogeny. This observation matches previous findings for character evolution in rhabditid nematodes which showed that homoplasy (convergent or parallel evolution) is a common theme in this group [2,50,51]. Importantly, our current study confirms that hermaphroditism evolved convergently in *C. briggsae *and *C. elegans*. Furthermore, we found that hermaphroditism evolved a third time in one of the new *Caenorhabditis *species, *C*. sp. 11.

### New *Caenorhabditis *species as a resource for future studies

The new *Caenorhabditis *species provide a phylogenetic framework to study the evolution of a number of genomic and phenotypic traits. At the molecular level, previously analyzed species were very distant from each other [3], and nucleotide turnover at putatively neutral sites was saturated, preventing the application of several molecular evolution tests. The new species provide several cases of more closely related species pairs, especially the *C. briggsae*/*C*. sp. 9 [52,53], *C. drosophilae*/*C*. sp. 2 and *C. angaria*/*C*. sp. 12 comparisons (Figure 2). In addition, the level of polymorphism within some of the new gonochoristic species is high, e.g. in *C*. sp. 5 where it is comparable to that of the ascidian *Ciona savignyi *[54]. Intraspecific genome comparisons in such cases are likely to reveal which parts of the genome are less constrained than other parts. Active genome sequencing is presently ongoing for the new *Caenorhabditis *species (see http://www.nematodes.org/nematodegenomes), and data are already available for *C*. spp. 7, 9 and 11 from the Genome Center at Washington University and GenBank, and *C*. sp. 5 from Genepool at the University of Edinburgh.

Some species provide interesting phenotypic features. For instance, *C*. sp. 11 provides a third example--after *C. elegans *and *C. briggsae*--of independent evolution of hermaphroditism from a gonochoristic ancestor. *C*. sp. 9 and *C. briggsae *are the first species pair in *Caenorhabditis *with partially fertile progeny, providing a genetic entry into species isolation studies [52,53]. These two species have different modes of reproduction (gonochoristic for *C*. sp. 9, hermaphroditic for *C. briggsae*), thus also allowing for genetic studies of reproductive mode evolution. Other species pairs, such as *C. angaria *and *C*. sp. 12, offer sterile hybrids of both sexes, and crosses of *C*. sp. 5 and *C. briggsae *yield sterile adult females with abnormal gonads (Additional File 1). Another example is sperm size, which is under selection in these nematodes [55,56]. Hermaphrodites have smaller sperm than males [57], males in hermaphroditic species have smaller sperm than males in gonochoristic species [57], and as a surprising extreme, *C*. sp. 18 males produce giant sperm (Additional File 13). These new species considerably widen the spectrum of phenotypic evolution that can be studied using *Caenorhabditis*.

## Authors' contributions

MAF collected a large number of strains used in this study and performed mating tests. KK collected molecular and morphological data. DHAF and KK performed the phylogenetic analyses. MAF and KK designed the study and wrote the text with the help of DHAF. MA, MVR, CB and JBP contributed to strain collection and species discovery. MA, MVR and JBP contributed to mating tests and MA to acquisition of sequence data. All authors read and approved the final manuscript.

## Supplementary Material

Additional file 1 **Crosses between strains and species**. A table showing the results of crosses between strains of the same species and of different species. Click here for file

Additional file 2 **Primer sequences**. Primer sequences for amplification and sequencing of 9 genes. Click here for file

Additional file 3 **Accession numbers**. GenBank Accession numbers of the sequences used in this study. Click here for file

Additional file 4 **Matrix of molecular sequence data**. Data matrix with concatenated sequences of 11 genes with additional information for maximum parsimony analysis. Click here for file

Additional file 5 ***Drosophila *RNAP2 sequences**. Alignment of RNA polymerase II sequences for 12 *Drosophila *species. Click here for file

Additional file 6 **Aligned ITS2 sequences**. Alignment of the region between the genes for 5.8S and LSU rRNA (ITS2) for 50 *Caenorhabditis *strains. Click here for file

Additional file 7 **New isolates of described *Caenorhabditis *species**. A table which lists strains of described *Caenorhabditis *species that were sampled from rotting plant material. Click here for file

Additional file 8 **Isolates of new *Caenorhabditis *species**. A table which lists strains of the new *Caenorhabditis *species with location and habitat. Click here for file

Additional file 9 ***Drosophila *RNAP2 phylogram**. Likelihood phylogram for Drosophila species calculated for RNA polymerase II (215 kD subunit) genes. Click here for file

Additional file 10 **Distribution of phenotypic characters**. Matrix for phenotypic characters and phylogeny. In MacClade or Mesquite, this file will provide a visualization of the distribution of the characters on the trees and the evolutionary changes. Click here for file

Additional file 11 **Differences in the ITS2 region**. Number and kind of differences in the ITS2 region between *Caenorhabditis *strains. Click here for file

Additional file 12 **Geographic distribution of previously known *Caenorhabditis *species used in this study**. Map showing the geographic distribution of *C. elegans *and *C. briggsae *and second map showing the distribution of previously known gonochoristic *Caenorhabditis *species. Click here for file

Additional file 13 **Large sperm in *C*. sp. 18**. Micrographs showing the large sperm size in *C*. sp. 18 compared to typical sperm size in *C*. sp. 17. Click here for file
