# Supplementary material for: A phylogeny and molecular barcodes for Caenorhabditis, with numerous new species from rotting fruits
Source: BMC Evol Biol. 2011 Nov 21;11:339. doi: 10.1186/1471-2148-11-339 (PMC3277298; doi:10.1186/1471-2148-11-339)
Supplement: Additional file 7 — New isolates of described Caenorhabditis species. A table which lists strains of described Caenorhabditis species that were sampled from rotting plant material. [file 1471-2148-11-339-S7.DOC]

**Described *Caenorhabditis* species sampled from rotting plant material.**

Further sampling of *C. elegans* and *C. briggsae* in France will be reported elsewhere (M.-A. Félix, unpublished).

| **Species** | **Substrate** | **Collector** | **Location**  **latitude, longitude** | **Time collected** | **Isolated by** | **Isolate** |
| --- | --- | --- | --- | --- | --- | --- |
| *brenneri* | rotting coconut | M.-A. Félix | Poovar, Kerala, India,  8.3087, 77.0807 | 19 Dec 2007 | M.-A. Félix | JU1323 |
| *brenneri* | rotting pineapple | M.-A. Félix | Poovar, Kerala, India  8.3087, 77.0807 | 19 Dec 2007 | M.-A. Félix | JU1324 |
| *brenneri* | rotting banana leaves and stems | M.-A. Félix | near Allepey, Kerala, India  9.6, 76.36 | 24 Dec 2007 | M.-A. Félix | JU1326 |
| *brenneri* | rotting pineapples | M.-A. Félix | Kanjirapally, Kerala, India  9.55, 76.8 | 26 Dec 2007 | M.-A. Félix | JU1327 |
| *brenneri* | fallen banana tree | M.-A. Félix | Kanjirapally, Kerala, India  9.55, 76.8 | 26 Dec 2007 | M.-A. Félix | JU1329 |
| *brenneri* | rotting *Alpinia purpurata* flower | V. Robert,  L. Sablé | La Réunion  -21.0476, 55.6905 | Jan 2008 | M.-A. Félix | JU1379 |
| *brenneri* | rotting noni fruits | - | next to Medellin, Colombia  5.9, -75.9 | Mar 2008 | M.-A. Félix | JU1396 |
| *brenneri* | rotting oranges | - | next to Medellin, Colombia  5.9, -75.9 | Mar 2008 | M.-A. Félix | JU1397 |
| *brenneri* | rotting oranges | - | next to Medellin, Colombia  5.9, -75.9 | Mar 2008 | M.-A. Félix | JU1398 |
| *briggsae* | rotting apricot | W. Davis | Salt Lake City, Utah, USA  40.7073, -111.8675 | 4 Aug 2006 | M. Ailion | EG4181 |
| *briggsae* | rotting pears | M.-A. Félix | Le Blanc, Indre, France  46.63, 1.06 | 15 Oct 2006 | M.-A. Félix | JU1038 |
| *briggsae* | leaf litter, bark, soil | M.-A. Félix | Kakegawa, Shizuoka Prefecture, Japan  34.7615, 138.0168 | 14 Mar 2007 | M.-A. Félix | JU1085 |
| *briggsae* | rotting plum | M.-A. Félix | Le Blanc, Indre, France  46.63, 1.06 | 11 Sep 2007 | M.-A. Félix | JU1205 |
| *briggsae* | rotting apple | M.-A. Félix | Santeuil, Val d'Oise, France  49.12618, 1.96152 | 14 Oct 2007 | M.-A. Félix | JU1254 |
| *briggsae* | rotting hawthorn fruits | M.-A. Félix | Santeuil, Val d'Oise, France  49.1255, 1.9510 | 14 Oct 2007 | M.-A. Félix | JU1257 |
| *briggsae* | rotting grapes | M.-A. Félix | Santeuil, Val d'Oise, France  49.12610, 1.95034 | 14 Oct 2007 | M.-A. Félix | JU1261 |
| *briggsae* | rotting plum | S. Jensen | Cottonwood Heights, Utah, USA  40.6, -111.8 | 17 Oct 2007 | M. Ailion | EG4360 |
| *briggsae* | rotting acorn | L. Hauth | Salt Lake City, Utah, USA  40.77, -111.83 | 21 Oct 2007 | M. Ailion | EG4365 |
| *briggsae* | rotting coconut | M.-A. Félix | Poovar, Kerala, India  8.32, 77.075 | 16 Dec 2007 | M.-A. Félix | JU1337 |
| *briggsae* | rotting palm fruits | M.-A. Félix | Trivandrum Botanical Garden, Kerala, India  8.509, 76.956 | 21 Dec 2007 | M.-A. Félix | JU1338 |
| *briggsae* | rotting wild *Ficus* fruits | M.-A. Félix | near Meenmutti Waterfall, Kerala, India  8.7119,77.1287 | 22 Dec 2007 | M.-A. Félix | JU1339 |
| *briggsae* | rotting parasitic flower and leaf litter | M.-A. Félix | Ponmudi, Kerala, India  8.76405, 77.11337 | 22 Dec 2007 | M.-A. Félix | JU1340 |
| *briggsae* | unidentified rotting red wild fruits | M.-A. Félix | Ponmudi, Kerala, India  8.7633, 77.114-5 | 22 Dec 2007 | M.-A. Félix | JU1341, JU1342 |
| *briggsae* | rotting large wild chestnut | M.-A. Félix | Ponmudi, Kerala, India  8.7625, 77.116 | 22 Dec 2007 | M.-A. Félix | JU1343 |
| *briggsae* | unidentified rotting wild fruits | M.-A. Félix | Ponmudi, Kerala, India  8.7629, 77.1155 | 22 Dec 2007 | M.-A. Félix | JU1344 |
| *briggsae* | fallen banana tree | M.-A. Félix | Kanjirapally, Kerala, India  9.55, 76.8 | 26 Dec 2007 | M.-A. Félix | JU1346 |
| *briggsae* | rotting *Etlingeria elatior* flowers | Mr. and Mrs Robert | La Réunion  -21.0476, 55.6905 | 22 Jan 2008 | M.-A. Félix | JU1377 |
| *briggsae* | rotting velvet apples (*Diospyros philippensis*) | Mr. and Mrs Robert | La Réunion  -21.0478, 55.6878 | 22 Jan 2008 | M.-A. Félix | JU1378 |
| *briggsae* | rotting *Couroupita guianensis* fruits | - | Parque de Flamengo, Rio de Janeiro, Brazil  -22.916, -43.173 | Jan 2008 | - | JU1392 |
| *briggsae* | rotting orange fruits | - | near Medellin, Colombia  5.9, -75.9 | Mar 2008 | M.-A. Félix | JU1399 |
| *briggsae* | unidentified rotting wild fruit | D. Baïlle | near Ba Be lake, Vietnam  22.4, 105.6 | 29 Apr 2008 | M.-A. Félix | JU1424 |
| *briggsae* | unidentified rotting fruit | M.-A. Moal | Saint-Gilles-les-Hauts, La Réunion  -21.05, 55.27 | May 2008 | M.-A. Félix | JU1435 |
| *briggsae* | rotting wild cherries and leaves | M.-A. Félix | Gif-sur-Yvette, Essonne, France  48.7069, 2.1296 | 26 Jun 2008 | M.-A. Félix | JU1467 |
| *briggsae* | rotting *Prunus* fruits | C. Braendle | Solothurn, Switzerland  47.1984, 7.53803 | 20 Jul 2008 | C. Braendle | NIC5 |
| *briggsae* | rotting peach | M.-A. Félix | Le Blanc; Indre, France  46.63, 1.06 | 18 Aug 2008 | M.-A. Félix | JU1496 |
| *briggsae* | rotting apple | M.-A. Félix | Le Blanc; Indre, France  46.63, 1.06 | 18 Aug 2008 | M.-A. Félix | JU1499 |
| *briggsae* | rotting crab apple | M. Leroy | Cold Spring Harbor, NY, USA  48.7006, 2.1804 | Aug 2008 | M. Leroy | JU1562 |
| *briggsae* | cabbage leaves and soil | M.-A. Félix | Valley de Torre, Santo Antao Island, Cape Verde  17.1363, -25.0682 | 26 Apr 2009 | M.-A. Félix | JU1637 |
| *briggsae* | soil next to taro/yam plant | M.-A. Félix | idem  17.13768, -25.06689 | 26 Apr 2009 | M.-A. Félix | JU1638 |
| *briggsae* | rotting jack fruit | - | near Dois Rios, Ilha Grande, Brazil  -23.18234, -44.19456 | 29 Apr 2009 | M. Ailion | EG5612 |
| *briggsae* | unidentified rotting wild fruit | - | near Dois Rios, Ilha Grande, Brazil  -23.18234, -44.19456 | 29 Apr 2009 | M. Ailion | EG5613 |
| *briggsae* | unidentified rotting wild flower | - | near Dois Rios, Ilha Grande, Brazil  -23.18234, -44.19456 | 29 Apr 2009 | M. Ailion | EG5614 |
| *briggsae* | rotting guava fruit | C. Gosse | Salazie, Ilot à Vidot, La Réunion  -21.0278, 55.5392 | 5 Jun 2009 | C. Braendle | NIC17 |
| *briggsae* | rotting tomatoes | M. Rockman | 6th Street at Jersey Ave, Jersey City, NJ, USA  40.7254194, -74.04515278 | 18 Jul 2009 | M. Rockman | QG107-QG121 |
| *briggsae* | unidentified rotting fallen flowers | M. Rockman | Hanalei Pavilion Beach Park, Kauai, Hawaii, USA  22.2065306, -159.4978222 | 2 Aug 2009 | M. Rockman | QG133 |
| *briggsae* | large rotting stone fruit | M. Rockman | Head of the Kalalau Trail, Kauai, Hawaii, USA  22.220225, -159.5829639 | 2 Aug 2009 | M. Rockman | QG128-QG130 |
| *briggsae* | large rotting stone fruit | M. Rockman | Kuilau Trail, Kauai, Hawaii, USA  22.0804444, -159.4124444 | 3 Aug 2009 | M. Rockman | QG132 |
| *briggsae* | rotting apricot | M. Ailion | Salt Lake City, Utah, USA  40.7073, -111.8675 | 19 Jul 2009 | M. Ailion | EG5715 |
| *briggsae* | rotting chestnut | D. Ailion | Moorea, French Polynesia  -17.53, -149.83 | 24 Jul 2009 | M. Ailion | EG6179 |
| *briggsae* | rotting apricot | W. Davis | Salt Lake City, Utah, USA  40.7073, -111.8675 | 28 Aug 2009 | M. Ailion | EG5784 |
| *briggsae* | unidentified rotting fruits | I. Nuez | Anse des Cascades, La Réunion  -21.12, 55.778 | 19 Sep 2009 | I. Nuez | JU1799 |
| *briggsae* | unidentified rotting fruits | V. Robert | Anse des Cascades, La Réunion  -21.12, 55.778 | Sep 2009 | I. Nuez | JU1802 |
| *briggsae* | rotting figs | C. Gosse | Yangmingshan, Taiwan  25.1588, 121.54 | 11 Nov 2009 | C. Braendle | NIC19 |
| *briggsae* | rotting figs | C. Gosse | Tai’an, Taiwan  24.3831, 121.0335 | 14 Nov 2009 | C. Braendle | NIC20 |
| *briggsae* | rotting leaves | C. Gosse | Yushan, Taiwan  23.4695, 120.9579 | 15 Nov 2009 | C. Braendle | NIC21 |
| *briggsae* | rotting *Datura* fruits | C. Gosse | Basianshan, Heping Township, Taiwan  24.1749, 120.8836 | 17 Nov 2009 | C. Braendle | NIC22 |
| *briggsae* | unidentified rotting wild fruit | Ms. Jung | National Reserve of Cerro San Gil, Guatemala  15.7, -88.8 | Dec 2009 | M.-A. Félix | JU1884 |
| *briggsae* | unidentified rotting fruits | C.Y. Kim | Kandy National park in Sri Lanka  7.2995, 80.6437 | 13 Dec 2009 | J.-B. Pénigault | JU1885 |
| *briggsae* | unidentified rotting fruits | F. Duveau | Millaa Millaa, Queensland, Australia  -17.495, 145.612 | Feb 2010 | M.-A. Félix | JU1907 |
| *briggsae* | unidentified rotting fruits (figs?) | F. Duveau | Millaa Millaa, Queensland, Australia  -17.495, 145.612 | Feb 2010 | M.-A. Félix | JU1908 |
| *briggsae* | unidentified rotting fruits | F. Duveau | Lucinda, Queensland, Australia  -18.5245, 146.3327 | Feb 2010 | M.-A. Félix | JU1909 |
| *briggsae* | rotting *Hibiscus* flowers | F. Duveau | Long Island (Whitsundays), Queensland, Australia  -20.37, 148.85 | Feb 2010 | F. Duveau | JU1912 |
| *briggsae* | unidentified rotting fruits | F. Duveau | Long Island (Whitsundays), Queensland, Australia  -20.37, 148.85 | Feb 2010 | F. Duveau | JU1913 |
| *briggsae* | unidentified rotting fruits | F. Duveau | Brisbane Botanical Garden, Queensland, Australia  -27.48, 153.03 | Mar 2010 | F. Duveau | JU1914 |
| *briggsae* | rotting breadfruit (*Artocarpus altilis*) | C. Braendle | Capesterre Belle-Eau, Guadeloupe  16.05, -61.57 | 17 Mar 2010 | C. Braendle | NIC115 |
| *briggsae* | rotting *Cecropia* fruits | C. Braendle | Saut de la Lezarde, Vernou, Guadeloupe  16.1793, -61.6558 | 10 Mar 2010 | C. Braendle | NIC116 |
| *briggsae* | unidentified rotting fruit | C. Braendle | Saut de la Lezarde, Vernou, Guadeloupe  16.1793, -61.6558 | 10 Mar 2010 | C. Braendle | NIC117 |
| *briggsae* | rotting banana stem | C. Braendle | Saut de la Lezarde, Vernou, Guadeloupe  16.1793, -61.6558 | 10 Mar 2010 | C. Braendle | NIC119 |
| *briggsae* | rotting *Cecropia* fruits | C. Braendle | Saut de la Lezarde, Vernou, Guadeloupe  16.1793,-61.6558 | 10 Mar 2010 | C. Braendle | NIC123 |
| *briggsae* | unidentified rotting fruit | M. Ailion | Cayey, Puerto Rico  18.11, -66.16 | 11 Mar 2010 | M. Ailion | EG6257 |
| *briggsae* | unidentified rotting stem | M. Ailion | Cayey, Puerto Rico  18.11, -66.16 | 11 Mar 2010 | M. Ailion | EG6258 |
| *briggsae* | rotting Christmas palm seed (*Veitchia merrillii*) | M. Ailion | Cayey, Puerto Rico  18.11, -66.16 | 11 Mar 2010 | M. Ailion | EG6259 |
| *briggsae* | unidentified rotting fruit | M. Ailion | Cayey, Puerto Rico  18.11, -66.16 | 11 Mar 2010 | M. Ailion | EG6260 |
| *briggsae* | rotting guava | M. Ailion | Cayey, Puerto Rico  18.11, -66.16 | 11 Mar 2010 | M. Ailion | EG6261 |
| *briggsae* | unidentified rotting seed | M. Ailion | Cayey, Puerto Rico  18.11, -66.16 | 11 Mar 2010 | M. Ailion | EG6262 |
| *briggsae* | rotting guava | M. Ailion | Cayey, Puerto Rico  18.11, -66.16 | 11 Mar 2010 | M. Ailion | EG6263 |
| *briggsae* | rotting banana flower | M. Ailion | Cayey, Puerto Rico  18.11, -66.16 | 12 Mar 2010 | M. Ailion | EG6264 |
| *briggsae* | unidentified rotting seed | Z. Negrón | Cayey, Puerto Rico  18.11, -66.16 | 12 Mar 2010 | M. Ailion | EG6265 |
| *briggsae* | unidentified rotting flower | M. Ailion | Cayey, Puerto Rico  18.11, -66.16 | 11 Mar 2010 | M. Ailion | EG6266 |
| *briggsae* | rotting fruit, probably *Terminalia catappa* | M. Ailion | Piñones, Puerto Rico  18.45, -66.0 | 16 Mar 2010 | M. Ailion | EG6267 |
| *briggsae* | unidentified rotting seed | M. Ailion | El Yunque, Puerto Rico  18.3, -65.8 | 14 Mar 2010 | M. Ailion | EG6268 |
| *briggsae* | unidentified rotting fruit | M. Ailion | El Yunque, Puerto Rico  18.3, -65.8 | 14 Mar 2010 | M. Ailion | EG6269 |
| *briggsae* | unidentified rotting seed | M. Ailion | El Yunque, Puerto Rico  18.3, -65.8 | 14 Mar 2010 | M. Ailion | EG6270 |
| *briggsae* | unidentified rotting seed | M. Ailion | El Yunque, Puerto Rico  18.3, -65.8 | 14 Mar 2010 | M. Ailion | EG6271 |
| *briggsae* | rotting fruit, probably *Terminalia catappa* | M. Ailion | Piñones, Puerto Rico  18.45, -66.0 | 16 Mar 2010 | M. Ailion | EG6272 |
| *briggsae* | unidentified rotting fruit | S. Dalton | El Yunque, Puerto Rico  18.3, -65.8 | 28 Mar 2010 | S. Dalton | EG6329 |
| *briggsae* | rotting orange | - | São Paulo, Brazil  -23.5, -46.7 | 13 May 2010 | M. Ailion | EG6273 |
| *briggsae* | wet moss | - | São Paulo, Brazil  -23.5, -46.7 | 13 May 2010 | M. Ailion | EG6274 |
| *briggsae* | rotting pitanga fruit | - | São Paulo, Brazil  -23.5, -46.7 | 13 May 2010 | M. Ailion | EG6275 |
| *briggsae* | rotting coconut | - | São Paulo, Brazil  -23.5, -46.7 | 13 May 2010 | M. Ailion | EG6276 |
| *briggsae* | unidentified rotting leaves | - | São Paulo, Brazil  -23.5, -46.7 | 13 May 2010 | M. Ailion | EG6313 |
| *elegans* | unidentified rotting fruit | M.-A. Félix | Botanical Garden, Lisbon, Portugal  38.7175, -9.1486 | 10 July 2005 | M.-A. Félix | JU775 |
| *elegans* | rotting *Ficus isophlebia* fruits | M.-A. Félix | Botanical Garden, Lisbon, Portugal  38.7190, -9.1491 | 10 July 2005 | M.-A. Félix | JU778 |
| *elegans* | rotting apples | M. Ailion | Salt Lake City, Utah, USA  40.77167, -111.87316 | 2 Oct 2006 | M. Ailion | EG4348-EG4352 |
| *elegans* | rotting tomatoes | M. Ailion | Eugene, Oregon, USA  44.04789, -123.07108 | 9 Oct 2006 | M. Ailion | EG4346, EG4347 |
| *elegans* | rotting plums | M.-A. Félix | Le Blanc, Indre, France  46.63, 1.06 | 15 Oct 2006 | M.-A. Félix | JU1026 |
| *elegans* | rotting pears | M.-A. Félix | Le Blanc, Indre, France  46.63, 1.06 | 15 Oct 2006 | M.-A. Félix | JU1039 |
| *elegans* | rotting apples | M. Ailion | Amares, Portugal  41.6288, -8.3476 | 28 Mar 2007 | M. Ailion | EG4724, EG4725 |
| *elegans* | rotting English walnuts | G. Hollopeter | Salt Lake City, Utah, USA  40.72596, -111.82184 | 27 Sep 2007 | M. Ailion | EG4945-EG4950 |
| *elegans* | rotting apples | W. Davis | Salt Lake City, Utah, USA  40.7073, -111.8675 | 4 Oct 2007 | M. Ailion | EG4680, EG4689 |
| *elegans* | rotting English walnuts | G. Hollopeter | Salt Lake City, Utah, USA  40.72596, -111.82184 | 9 Oct 2007 | M. Ailion | EG4951-EG4957 |
| *elegans* | rotting apple | M.-A. Félix | Santeuil, Val d'Oise, France  49.1269, 1.9595 | 14 Oct 2007 | M.-A. Félix | JU1218 |
| *elegans* | rotting oranges | M.-A. Félix | Garden Catalina de Ribera, Sevilla, Spain  37.3845, -5.988 | 29 Mar 2008 | M.-A. Félix | JU1400 |
| *elegans* | rotting *Opuntia ficus-indica* cactus fruit | M.-A. Félix | Carmona, Spain  37.468, -5.637 | 31 Mar 2008 | M.-A. Félix | JU1409 |
| *elegans* | rotting palm fruits | M.-A. Félix | Park Guëll, Barcelona, Spain  41.41307, 2.15231 | 9 Jun 2008 | M.-A. Félix | JU1440 |
| *elegans* | rotting palm fruits (*Chamaerops humilis*) | C. Braendle | Endoume, Marseille, France  43.2792, 5.3543 | 14 Sep 2008 | C. Braendle | NIC1 |
| *elegans* | rotting figs | C. Braendle | L'Estaque, Marseille, France  43.3656, 5.3056 | 28 Sep 2008 | C. Braendle | NIC3 |
| *elegans* | rotting sloe fruits | M.-A. Félix | Plougasnou, Finistère, France  48.7050, -3.7952 | 14 Jul 2009 | M.-A. Félix | JU1666 |
| *elegans* | rotting solanaceous fruit | M. Rockman | Buena Vista Park, San Francisco, CA  37.7679194 -122.4414056 | 28 Dec 2010 | M. Rockman | QG535-QG539 |
| *elegans* | rotten orange peel | A. Paaby | State St., Santa Barbara, CA  34.421629, -119.702021 | June 2011 | A. Paaby | QG558 |
| *elegans* | soil under rotting fruit | M. Rockman | Jersey St. San Francisco, CA  37.7501, -122.433 | 26 Nov 2007 | M. Rockman | QX1211 |
| *remanei* | rotting apple | M.-A. Félix | Obernai, Bas-Rhin, France  48.4602, 7.47012 | 2 Oct 2005 | M.-A. Félix | JU825 |
| *remanei* | rotting fruits and vegetables | M.-A. Félix | Okazaki, Aichi Prefecture, Japan  34.9538, 137.1723 | 11 Mar 2007 | M.-A. Félix | JU1082 |
| *remanei* | rotting fruits and vegetables | M.-A. Félix | Kakegawa, Shizuoka Prefecture, Japan  34.7622, 138.0144 | 14 Mar 2007 | M.-A. Félix | JU1084 |
| *remanei* | leaf litter, bark, soil | M.-A. Félix | Botanical Gardens, University of Tokyo, Japan  35.69, 139.69 | 18 Mar 2007 | M.-A. Félix | JU1087 |
| *remanei* | rotting crab apple | M. Leroy | Cold Spring Harbor, NY, USA  40.86, -73.47 | Aug 2008 | M. Leroy | JU1565 |
| *remanei* | soil in eggplant field | J. Wang | Jiufeng Village, Wuhan City, Hubei Province, China  30.3051, 114.2942 | 27 Octo 2009 | J. Wang | VX0088 |
