# Supplementary material for: A phylogeny and molecular barcodes for Caenorhabditis, with numerous new species from rotting fruits
Source: BMC Evol Biol. 2011 Nov 21;11:339. doi: 10.1186/1471-2148-11-339 (PMC3277298; doi:10.1186/1471-2148-11-339)
Supplement: Additional file 8 — Isolates of new Caenorhabditis species. A table which lists strains of the new Caenorhabditis species with location and habitat. [file 1471-2148-11-339-S8.DOC]

**Isolates of new *Caenorhabditis* species.**

| **Species** | **Substrate** | **Collector** | **Location**  **latitude, longitude** | **Time collected** | **Isolated by** | **Isolate** |
| --- | --- | --- | --- | --- | --- | --- |
| sp. 5 | soil next to unidentified fruits | M.-A. Félix | Chengyang, Guangxi, China  25.902, 109.6395 | 6 May 2005 | M.-A. Félix | JU727 |
| sp. 5 | soil in garden | R. Radeck | Guangzhou, China  23.1, 113.3 | Jul 2005 | W. Sudhaus | SB378 |
| sp. 5 | unidentified rotting fruit | J. Milloz | Garden of Harmony, Suzhou, China  31.307, 120.624 | 17 Aug 2007 | J. Milloz | JU1201 |
| sp. 5 | same unidentified rotting fruit | J. Milloz | Feilaifeng park, Hangzhou, China  30.254, 120.160 | 25 Aug 2007 | J. Milloz | JU1202 |
| sp. 5 | unidentified rotting fruit | D. Baïlle | near Ba Be Lake, 105°35”E; 22°25”N, Vietnam  22.4, 105.6 | 29 Apr 2008 | M.-A. Félix | JU1423 |
| sp. 6 | rotting apples | M. Ailion, A.-J. Rodrigues | Amares, Portugal.  41.6288, -8.3476 | 28 Mar 2007 | M. Ailion | EG4788 |
| sp. 7 | rotting citrus fruit | M. Herrmann | Begoro, Ghana  6.38, -0.38 | Jun 2007 | M.-A. Félix | JU1199 |
| sp. 7 | leaf litter near pond edge | J. and G. Hatty | near Shonga, Nigeria  9.1, 5.1 | 29 Nov 2008 | M.-A. Félix | JU1593 |
| sp. 8 | rotting tomatoes | M. Rockman | Backyard of 93 Mercer St., Jersey City, NJ, USA  40.7187444, -74.04661667 | 28 Jul 2007 | M. Rockman | QX1182 |
| sp. 8 | rotting apple | D. Fitch | Newburgh, NY, USA  41.5724, -74.0261 | Aug 2007 | D. Fitch | DF5106 |
| sp. 8 | rotting peach | K. Kiontke | La Guardia Community Garden, New York City, USA  40.7276, -73.9991 | Aug 2010 | K. Kiontke | DF5115 |
| sp. 8 | rotting persimmon | K. Kiontke | Liz Christie Community Garden  New York City, USA  40.7236, -73.9907 | Aug 2010 | K. Kiontke | DF5116 |
| sp. 8 | rotting melon rind | L. Price | Fort Worth, TX, USA  32.63025, -97.398 | Sep 2007 | L. Price | APS1 |
| sp. 9 | rotting flowers and leaves | M.-A. Félix | Trivandrum Zoo, Kerala, India  8.512, 76.955 | 21 Dec 2007 | M.-A. Félix | JU1325 |
| sp. 9 | worked soil containing organic material | J. Ehrenkranz | Shinkolobwe, Katanga province, DR of Congo  -11, 26 | 20 May 2008 | T. Brock and M. Ailion | EG5268 |
| sp. 10 | rotting banana stem and leaf | M.-A. Félix | Kanjirapally, Kerala, India  9.55, 76.8 | 26 Dec 2007 | M.-A. Félix | JU1328 |
| sp. 10 | rotting nutmeg | M.-A. Félix | Kanjirapally, Kerala, India  9.55, 76.8 | 26 Dec 2007 | M.-A. Félix | JU1330 |
| sp. 10 | rotting bilimbi | M.-A. Félix | Kanjirapally, Kerala, India  9.55, 76.8 | 26 Dec 2007 | M.-A. Félix | JU1331 |
| sp. 10 | mix of coffee grains, soil and elephant dung | M.-A. Félix | Angela Spice Garden next to Periyar, Kerala, India  9.60, 77.13 | 27 Dec 2007 | M.-A. Félix | JU1332 |
| sp. 10 | rotting cacao fruit | M.-A. Félix | Angela Spice Garden, Kerala, India  9.60, 77.13 | 27 Dec 2007 | M.-A. Félix | JU1333 |
| sp. 10 | rotting red fruits | M.-A. Félix | Angela Spice Garden, Kerala, India  9.60, 77.13 | 27 Dec 2007 | M.-A. Félix | JU1334 |
| sp. 11 | rotting *Etlingeria elatior* flowers | V. Robert, L. Sablé | Saint-Benoît, La Réunion  -21.0473, 55.6885 | Jan 2008 | M.-A. Félix | JU1373 |
| sp. 11 | surface of a millipede in a sample of rotting banana flowers | V. Robert, L. Sablé | Saint-Benoît, La Réunion  -21.0484, 55.6878 | Jan 2008 | M.-A. Félix | JU1374 |
| sp. 11 | rotting *Duguetia surinamensis* fruit | P. Châtelet | Nouragues, French Guyana  4.08, -52.68 | May 2008 | M.-A. Félix | JU1428 |
| sp. 11 | rotting *Duguetia surinamensis* fruit | P. Châtelet | Nouragues Forest, French Guyana  4.08, -52.68 | May 2008 | M.-A. Félix | JU1429 |
| sp. 11 | rotting *Duguetia surinamensis* fruit | P. Châtelet | Nouragues Forest, French Guyana  4.08, -52.68 | May 2008 | M.-A. Félix | JU1430 |
| sp. 11 | rotting small tomatoes | M.-A. Félix | Matinho, Santo Antão Island, Cape Verde  17.1064, -25.1222 | 21 Apr 2009 | M.-A. Félix | JU1630 |
| sp. 11 | rotting tomatoes | M.-A. Félix | Caibros, Santo Antão Island, Cape Verde  17.1404, -25.1261 | 21 Apr 2009 | M.-A. Félix | JU1631 |
| sp. 11 | rotting *Cucurmis* fruit | M.-A. Félix | Boca de Ambas, Santo Antão Island, Cape Verde  17.1508, -25.1314 | 22 Apr 2009 | M.-A. Félix | JU1632 |
| sp. 11 | rotting coffee grains | M.-A. Félix | close to Cha de Fazenda, Santo Antão Island, Cape Verde  17.115, -25.05 | 24 Apr 2009 | M.-A. Félix | JU1633 |
| sp. 11 | rotting banana stem | M.-A. Félix | close to Cha de Padre, Santo Antão Island, Cape Verde  17.12, -25.049 | 24 Apr 2009 | M.-A. Félix | JU1634 |
| sp. 11 | rotting banana stem | M.-A. Félix | close to Cha de Padre on Santo Antão Island, Cape Verde  17.121, -25.0492 | 24 Apr 2009 | M.-A. Félix | JU1635 |
| sp. 11 | rotting banana stem | M.-A. Félix | between Corda and Cha de Coelho, Santo Antão Island, Cape Verde  17.129, -25.0966 | 25 Apr 2009 | M.-A. Félix | JU1636 |
| sp. 11 | rotting banana stem | M.-A. Félix | close to Xôxô, Santo Antão Island, Cape Verde  17.149, -25.0696 | 26 Apr 2009 | M.-A. Félix | JU1639 |
| sp. 11 | rotting tomato | M.-A. Félix | Ribeira Cumba, Santiago Island, Cape Verde  15.084, -23.5215 | 1 May 2009 | M.-A. Félix | JU1640 |
| sp. 11 | rotting banana and mango leaves | M.-A. Félix | Cidade Velha, Santiago Island, Cape Verde  14.9174, -23.6049 | 3 May 2009 | M.-A. Félix | JU1641 |
| sp. 11 | rotting *Pandanus* fruit | M. Rockman | Trail to Sealodge Beach, Kauai, Hawaii, USA  22.22955, -159.4764 | 3 Aug 2009 | M. Rockman | QG131 |
| sp. 11 | rotting *Manilkara bidentata* fruit | S. Dalton | El Yunque, Puerto Rico  18.3, -65.8 | 12 Oct 2009 | M. Ailion | EG5889 |
| sp. 11 | unidentified rotting flower | C. Braendle | Saut de la Lezarde, Vernou, Guadeloupe  16.1793, -61.6558 | 10 Mar 2010 | C. Braendle | NIC122 |
| sp. 11 | rotting *Cecropia* fruit | C. Braendle | Capesterre Belle-Eau, Guadeloupe  16.05, -61.57 | 17 Mar 2010 | C. Braendle | NIC114 |
| sp. 11 | unidentified rotting fruit | C. Braendle | Capesterre Belle-Eau, Guadeloupe  16.05, -61.57 | 17 Mar 2010 | C. Braendle | NIC120 |
| sp. 11 | unidentified rotting fruit | C. Braendle | Capesterre Belle-Eau, Guadeloupe  16.05, -61.57 | 17 Mar 2010 | C. Braendle | NIC121 |
| sp. 11 | rotting *Cecropia* fruit | C. Braendle | Capesterre Belle-Eau, Guadeloupe  16.05, -61.57 | 17 Mar 2010 | C. Braendle | NIC124 |
| sp. 11 | rotting carambola fruit | C. Braendle | Capesterre Belle-Eau, Guadeloupe  16.05, -61.57 | 17 Mar 2010 | C. Braendle | NIC125 |
| sp. 11 | unidentified rotting wild berry | M. Ailion | El Yunque, Puerto Rico  18.3, -65.8 | 14 Mar 2010 | M. Ailion | EG6180 |
| sp. 11 | unidentified rotting fruit | - | Amazon village, Brazil  -2.96, -59.84 | 22 Jun 2010 | M.-A. Félix | JU1975 |
| sp. 11 | unidentified rotting fruit | - | Amazon village, Brazil  -2.96, -59.84 | 22 Jun 2010 | M.-A. Félix | JU1976 |
| sp. 11 | rotting stem of *Heliconia bilhai* | N. Poullet | Capesterre Belle-Eau, Guadeloupe  16.05, -61.57 | 31 Jul 2010 | N. Poullet | NIC129 |
| sp. 12 | rotting *Micropholis cayennensis* fruit | P. Châtelet | Nouragues Forest, French Guyana  4.08, -52.68 | May 2008 | M.-A. Félix | JU1426 |
| sp. 12 | rotting *Micropholis cayennensis* fruit | P. Châtelet | Nouragues Forest, French Guyana  4.08, -52.68 | May 2008 | M.-A. Félix | JU1427 |
| sp. 13 | rotting apple | M.-A. Félix | Orsay apple orchard  48.7015, 2.1725 | 9 Sep 2008 | M.-A. Félix | JU1528 |
| sp. 13 | rotting apple | M.-A. Félix | Orsay apple orchard  48.7015, 2.1725 | 8 Nov 2010 | M.-A. Félix | JU2010 |
| sp. 14 | rotting (local) chestnut | D. Ailion | Moorea, French Polynesia  -17.53, -149.83 | 24 Jul 2009 | M. Ailion | EG5716 |
| sp. 14 | rotting coconut | José Gomes | Petit Bourg, Guadeloupe  16.185, -61.595 | Feb 2010 | M.-A. Félix | JU1905 |
| sp. 14 | rotting seeds | C. Braendle | Saut de la Lezarde, Vernou, Guadeloupe  16.1793, -61.6558 | 10 Mar 2010 | C. Braendle | NIC118 |
| sp. 14 | rotting soursoup fruits | N. Poullet | Capesterre Belle-Eau, Guadeloupe  16.05, -61.57 | 31 Jul 2010 | N. Poullet | NIC126 |
| sp. 14 | rotting breadfruit (*Artocarpus altilis*) | N. Poullet | Capesterre Belle-Eau, Guadeloupe  16.05, -61.57 | 31 Jul 2010 | N. Poullet | NIC127 |
| sp. 14 | rotting papaya fruits | N. Poullet | Capesterre Belle-Eau, Guadeloupe  16.05, -61.57 | 31 Jul 2010 | N. Poullet | NIC128 |
| sp. 15 | rotting fallen *Hibiscus* flowers | M. Rockman | Near Hanalei Bay, Kauai, Hawaii, USA  22.2022167, -159.511183 | 2 Aug 2009 | M. Rockman | QG122 |
| sp. 15 | unidentified rotting flowers | M. Rockman | Hanalei Pavilion Beach Park, Kauai, Hawaii, USA  22.2065306, -159.4978222 | 2 Aug 2009 | M. Rockman | QG123 |
| sp. 16 | rotting cacao fruit | J.-B. Pénigault | Sanda Center, Bali, Indonesia  -8.36130, 115.02965 | 11 Nov 2009 | J.-B. Pénigault | JU1873 |
| sp. 17 | rotting *Barokia* sp. fruit | C. Braendle, M.-A. Félix | Nouragues Forest, French Guyana  4.088017, -52.6751 | 21 Nov 2009 | C. Braendle, M.-A. Félix | JU1825 |
| sp. 17 | rotting Clusia grandiflora fruits | C. Braendle, M.-A. Félix | forest of Montagne des Singes, French Guyana  5.0692, -52.7113 | 27 Nov 2009 | C. Braendle, M.-A. Félix | NIC59 |
| sp. 18 | rotting *Hyeronima taxiflora* fruits | C. Braendle, M.-A. Félix | Nouragues Forest, French Guyana  4.091383, -52.68565 | 23 Nov 2009 | C. Braendle, M.-A. Félix | JU1857 |
| sp. 19 | rotting *Manilkara bidentata* fruit | S. Dalton | El Yunque, Puerto Rico  18.3, -65.8 | 28 Mar 2010 | S. Dalton, M. Ailion | EG6142 |
| sp. 20 | rotten *Heliconia* flowers | C. Braendle | Soufrière Forest trail, Guadeloupe  16.0328, -61.676 | Mar 2010 | C. Braendle | NIC113 |
