# Supplementary material for: A phylogeny and molecular barcodes for Caenorhabditis, with numerous new species from rotting fruits
Source: BMC Evol Biol. 2011 Nov 21;11:339. doi: 10.1186/1471-2148-11-339 (PMC3277298; doi:10.1186/1471-2148-11-339)
Supplement: Additional file 9 — Drosophila RNAP2 phylogram. Likelihood phylogram for Drosophila species calculated for RNA polymerase II (215 kD subunit) genes. [file 1471-2148-11-339-S9.PDF]

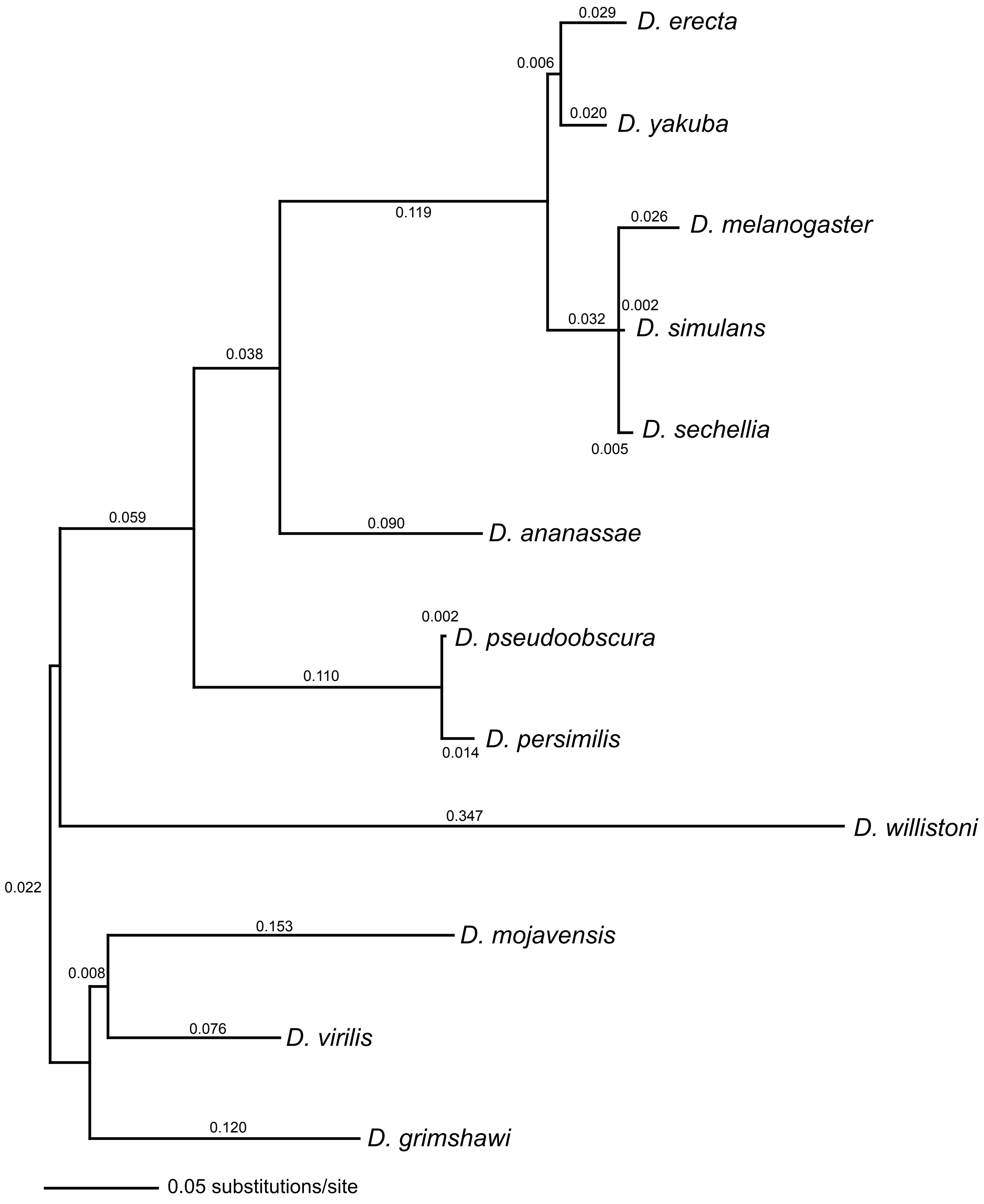

Likelihood phylogram for *Drosophila* species calculated for RNA polymerase II (215kD subunit) genes using a general time-reversible substitution model for which all parameters were estimated from the data. Topology from Stark et al. 2007; Nature 450; 219-232.
