# Supplementary material for: A phylogeny and molecular barcodes for Caenorhabditis, with numerous new species from rotting fruits
Source: BMC Evol Biol. 2011 Nov 21;11:339. doi: 10.1186/1471-2148-11-339 (PMC3277298; doi:10.1186/1471-2148-11-339)
Supplement: Additional file 11 — Differences in the ITS2 region. Number and kind of differences in the ITS2 region between Caenorhabditis strains. [file 1471-2148-11-339-S11.DOC]

Differences in the ITS2 sequences between *Caenorhabditis* strains

| species | strains | number of differences  (substitutions/indels) | kinds of substitution |
| --- | --- | --- | --- |
| *C. brenneri* | SB129 vs. LKC28 | 0/0 | -- |
|  | SB129, LKC28 vs. CB5161 | 2/1 | 1K, 1Y |
|  | SB129, LKC28 vs. SB280^1^ | 1/0 | 1R |
|  | SB280^1^ vs. CB5161 | 2/0 | 1K, 1R |
| *C. briggsae* | AF16 vs. QG129 | 0/0 | -- |
|  | QG133 vs. AF16, QG129 | 3/3 | 1K, 2R |
| *C.elegans* | N2 vs. LKC34 | 0/0 | -- |
|  | QG558 vs. QX1211 | 0/0 | -- |
|  | N2, LKC34 vs. QG558, QX1211 | 1/2 | 1W |
| *C. japonica* | DF5081 vs. SB339 | 0/0 | -- |
| *C. remanei* | PB206 vs. SB146 vs. VT733 | 0/0 | -- |
|  | JU724 vs. VX0088 | 0/0 | -- |
|  | PB206, SB146, VT733 vs. JU724, VX0088 | 11/1 | 1K, 5R, 1S, 2W, 2Y |
|  | PB206, SB146, VT733 vs. EM464 | 10/1 | 1K, 1M, 3R, 4W, 1Y |
|  | JU724, VX0088 vs. EM464 | 12/0 | 2K, 2R, 1S, 5W, 2Y |
| *C. angaria* | PS1010 vs. RGD1 | 0/0 | -- |
| *C.* sp. 5 | SB378 vs. JU727 | 1/0 | 1Y |
| *C.* sp. 8 | DF5106 vs. DF5115 | 0/0 | -- |
|  | DF5106, DF5115 vs. QX1182 | 23/4  2 indels larger than 1nt | 3K, 1M, 5R, 3S, 4W, 7Y |
|  | APS1^2^ vs. QX1182 | 17/5 plus unknown number of changes | 3K, 1R, 3S, 2W, 7Y |
|  | DF5106, DF5115 vs. APS1^2^ | 7/2 plus unknown number of changes | 3K, 1R, 2W, 1Y |
| *C.* sp. 9 | EG5268 vs. JU1325 | 0/0 | -- |
| *C.* sp. 11 | JU1428 vs JU1373 | 3/1 | 2M, 1W |
|  | QG131 vs JU1373 | 4/0 | 2M, 1R, 1W |
|  | QG131 vs JU1428 | 1/1 | 1R |
| *C.* sp. 14 | JU1905 vs EG5716^3^ | 0/0 | -- |
| *C.* sp. 17 | NIC59 vs JU1825 | 1/0 | 1Y |
| *C.* sp. 18 | JU1853 vs JU1857 | 0/0 | -- |

^1^ 10 nt not sequenced at 5'end; ^2^ large part of unknown size missing; ^3^ 107 nt not sequenced
