# Supplementary material for: A phylogeny and molecular barcodes for Caenorhabditis, with numerous new species from rotting fruits
Source: BMC Evol Biol. 2011 Nov 21;11:339. doi: 10.1186/1471-2148-11-339 (PMC3277298; doi:10.1186/1471-2148-11-339)
Supplement: Additional file 12 — Geographic distribution of previously known Caenorhabditis species used in this study. Map showing the geographic distribution of C. elegans and C. briggsae and second map showing the distribution of previously known gonochoristic Caenorhabditis species. [file 1471-2148-11-339-S12.PPT]

## Slide 1
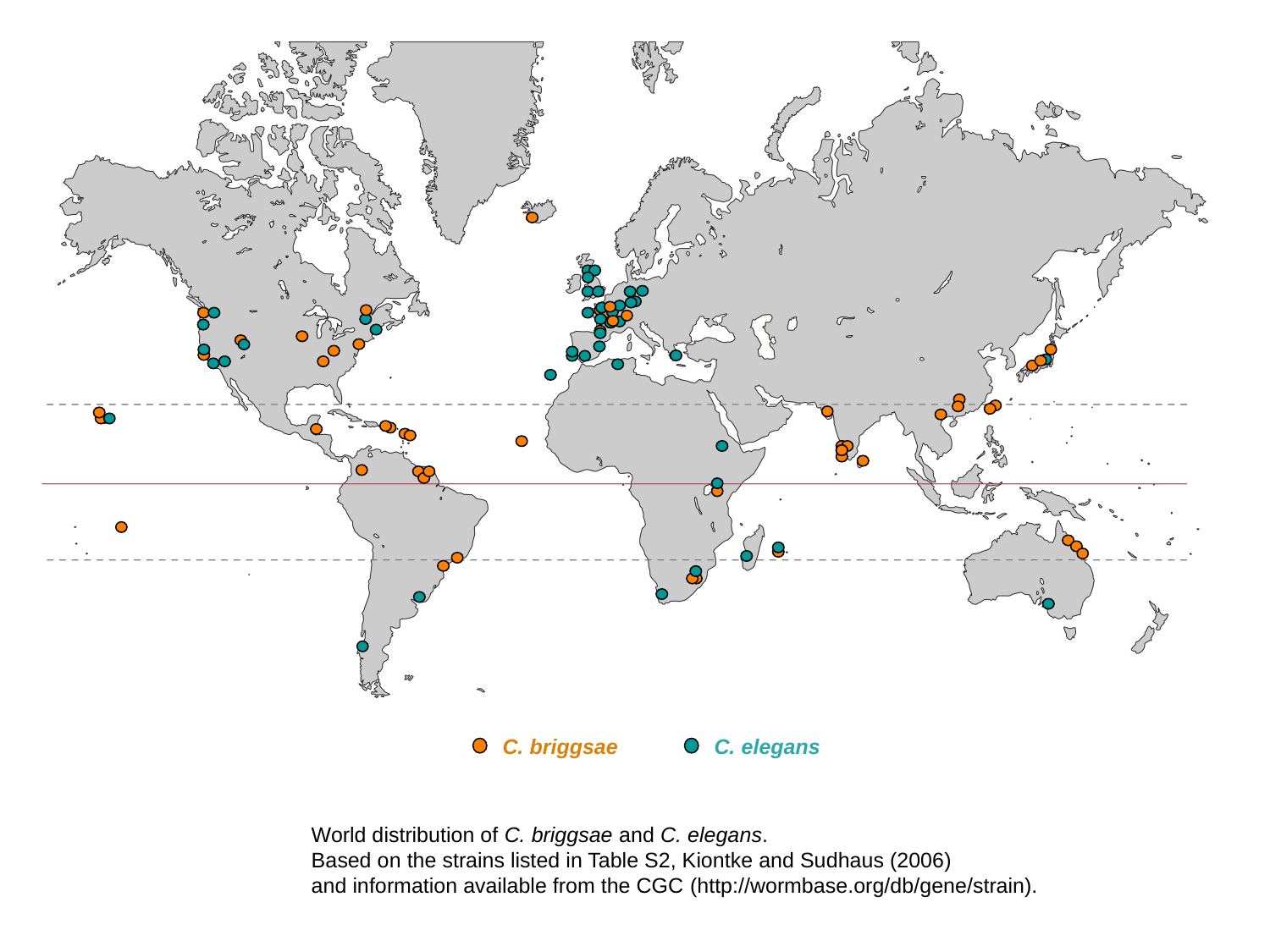

C. briggsae
C. elegans
World distribution of C. briggsae and C. elegans.
Based on the strains listed in Table S2, Kiontke and Sudhaus (2006)
and information available from the CGC (http://wormbase.org/db/gene/strain).

## Slide 2
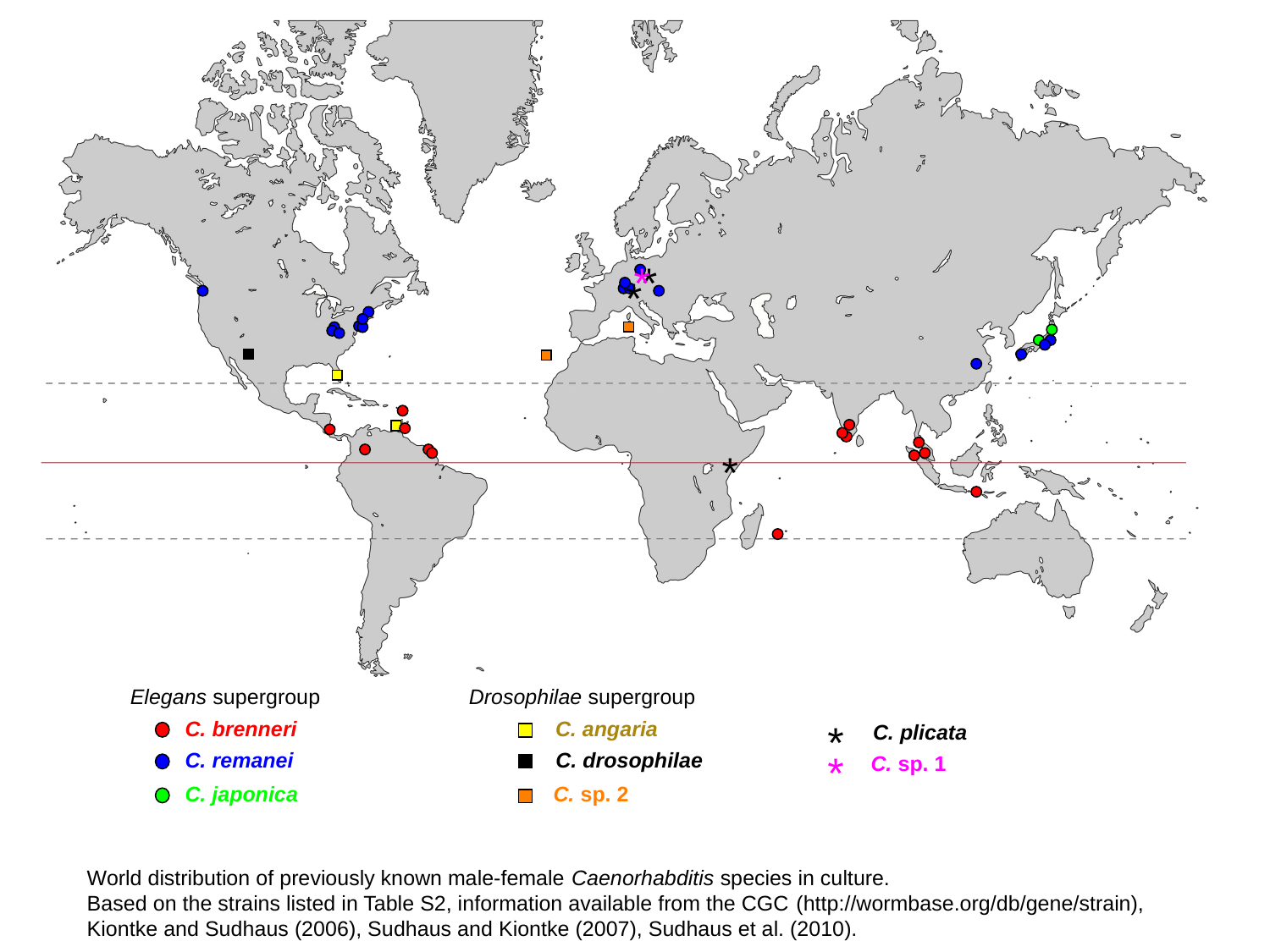

*
*
*
*
Elegans supergroup
Drosophilae supergroup
C. brenneri
C. angaria
*
C. plicata
*
C. remanei
C. drosophilae
C. sp. 1
C. japonica
C. sp. 2
World distribution of previously known male-female Caenorhabditis species in culture.
Based on the strains listed in Table S2, information available from the CGC (http://wormbase.org/db/gene/strain),
Kiontke and Sudhaus (2006), Sudhaus and Kiontke (2007), Sudhaus et al. (2010).
