# Supplementary material for: A phylogeny and molecular barcodes for Caenorhabditis, with numerous new species from rotting fruits
Source: BMC Evol Biol. 2011 Nov 21;11:339. doi: 10.1186/1471-2148-11-339 (PMC3277298; doi:10.1186/1471-2148-11-339)
Supplement: Additional file 13 — Large sperm in C. sp. 18. Micrographs showing the large sperm size in C. sp. 18 compared to typical sperm size in C. sp. 17. [file 1471-2148-11-339-S13.PPT]

## Slide 1
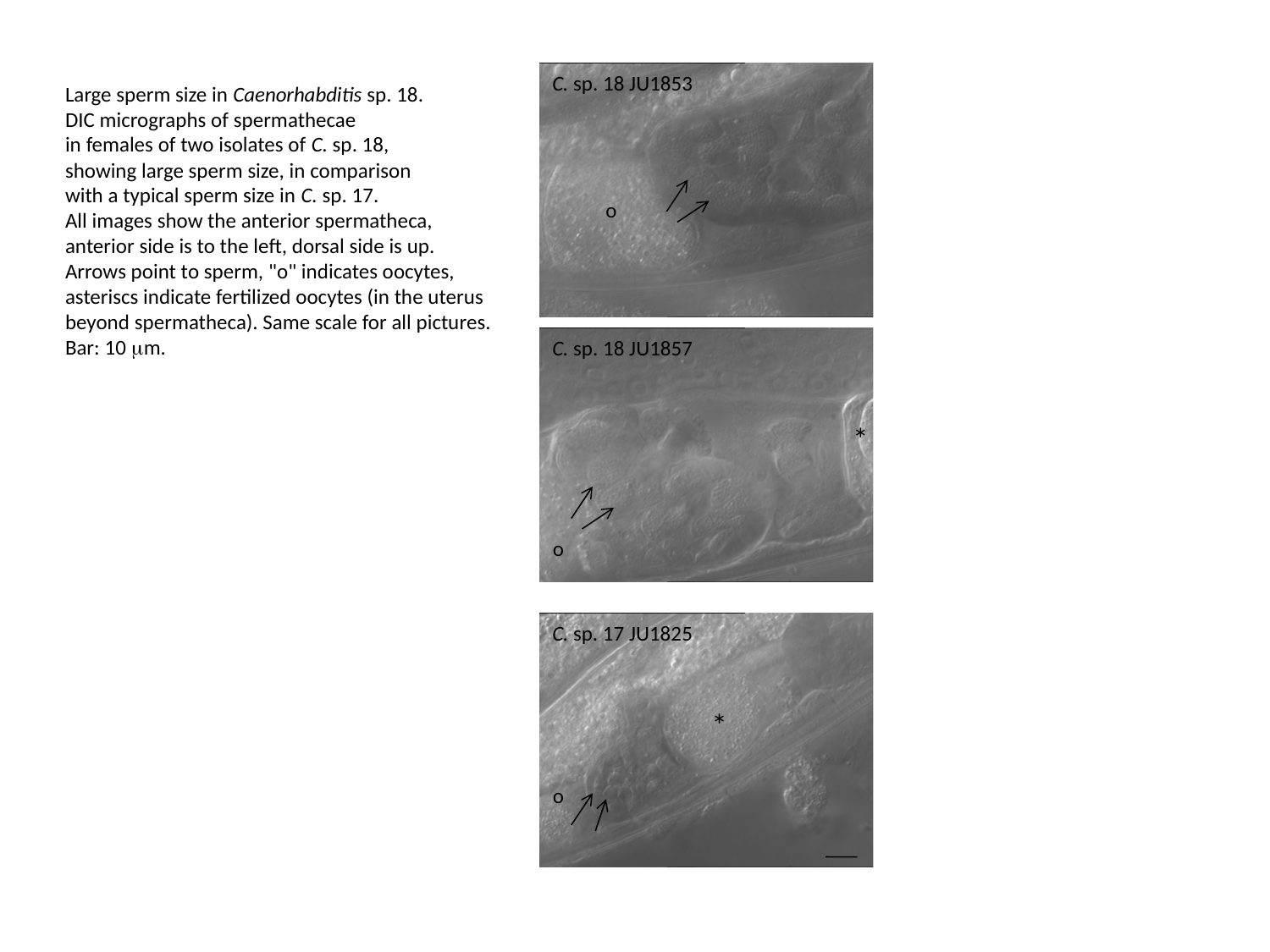

C. sp. 18 JU1853
Large sperm size in Caenorhabditis sp. 18.
DIC micrographs of spermathecae
in females of two isolates of C. sp. 18,
showing large sperm size, in comparison
with a typical sperm size in C. sp. 17.
All images show the anterior spermatheca, anterior side is to the left, dorsal side is up. Arrows point to sperm, "o" indicates oocytes,
asteriscs indicate fertilized oocytes (in the uterus beyond spermatheca). Same scale for all pictures. Bar: 10 μm.
o
C. sp. 18 JU1857
*
o
C. sp. 17 JU1825
*
o
